# Ferroelectric Material in Triboelectric Nanogenerator

**DOI:** 10.3390/ma17122834

**Published:** 2024-06-10

**Authors:** Zhiyu Zhang, Tong Wu, Enqi Sun, Yahui Chen, Ning Wang

**Affiliations:** 1 Center for Green Innovation, School of Mathematics and Physics, University of Science and Technology Beijing, Beijing 100083, China; 2 National Institute of Metrology China, National Institute of Metrology, Beijing 100029, China

**Keywords:** triboelectric nanogenerator, ferroelectric material, energy harvesting, multifunctionality, self-powered system

## Abstract

Ferroelectric materials, with their spontaneous electric polarization, are renewing research enthusiasm for their deployment in high-performance micro/nano energy harvesting devices such as triboelectric nanogenerators (TENGs). Here, the introduction of ferroelectric materials into the triboelectric interface not only significantly enhances the energy harvesting efficiency, but also drives TENGs into the era of intelligence and integration. The primary objective of the following paper is to tackle the newest innovations in TENGs based on ferroelectric materials. For this purpose, we begin with discussing the fundamental idea and then introduce the current progress with TENGs that are built on the base of ferroelectric materials. Various strategies, such as surface engineering, either in the micro or nano scale, are discussed, along with the environmental factors. Although our focus is on the enhancement of energy harvesting efficiency and output power density by utilizing ferroelectric materials, we also highlight their incorporation in self-powered electronics and sensing systems, where we analyze the most favorable and currently accessible options in attaining device intelligence and multifunctionality. Finally, we present a detailed outlook on TENGs that are based on ferroelectric materials.

## 1. Introduction

Petroleum, natural gas, and coal are examples of fossil fuels that have historically been important sources of energy worldwide [1,2]. The creation and utilization of these energy sources have culminated in pollution of the air and water, as well as greenhouse gas emissions that worsen climate change [3,4,5,6]. Apart from that, looking for renewable energy sources like solar, wind, hydro, and biomass has increased in relevance as an alternative for traditional fossil fuels as the world’s need for clean, sustainable energy resources progressively rises [7].

The convergence of the Internet of Things (IoT) and artificial intelligence (AI) are both growing at an astounding rate, and, consequently, portable electronics are becoming more and more necessary in our daily lives [8,9]. These little and potent gadgets, which range from wearables to smart homes, from smartphones to smartwatches, not only alter the way we live but also propel society’s technical advancement and dictate technology’s direction [10,11]. But the swift development of technology additionally made the need for sustainable electricity sources more urgent than ever. The rising reliance on power sources is a result of the increased use of portable electronic gadgets [12]. The resulting issue is that conventional power supply techniques frequently depend on finite resources, such as minerals and fossil fuels, and as a result can negatively impact the environment both during production and disposal. Discovering more environmentally friendly, renewable, high-performance, and energy-efficient power sources is essential for satisfying the demands of sustainable development [13].

Triboelectric nanogenerators (TENGs) are energy harvesting apparatus that were invented by Wang’s group in 2012 on the basis of the coupled effects of triboelectrification and electrostatic induction [14]. In actuality, the electrons on the surfaces of any com-bination of materials will transfer and separate as a result of the differing electron affinities of their surfaces when they come into contact to cause relative motion; this phenomenon is known in physics as the triboelectric effect [15]. Since then, various triboelectrically active materials such as polytetrafluoroethylene (PTFE) [16], cellulose [17], polyvinylidene fluoride (PVDF) [18], polydimethylsiloxane (PDMS) [19], and other friction materials [20,21] have been employed in the development of high-performance TENGs for gathering energy from wind, water flow, human movement (slow strolling, fast running, etc.), vibration, and other sources. Their advantages—miniaturization, energy self-sufficiency, environmental protection, and affordability—open up new avenues for the advancement and creativity of electronic gadgets in the future. Nevertheless, for keeping up with the increasing desire for microenergy harvesting and consumer technologies, TENGs need to overcome their intrinsic high inner resistance and low power density, especially in light of the quick development of the global Internet of Things (IoT) and wearable gadgets that are electronic [22,23]. Consequently, an increasing number of studies are trying to identify superior triboelectric materials, which is the basis and starting point of designing high-performance TENGs; although, other strategies such as structure design and surface engineering are also effective [24,25].

**Figure 1:**
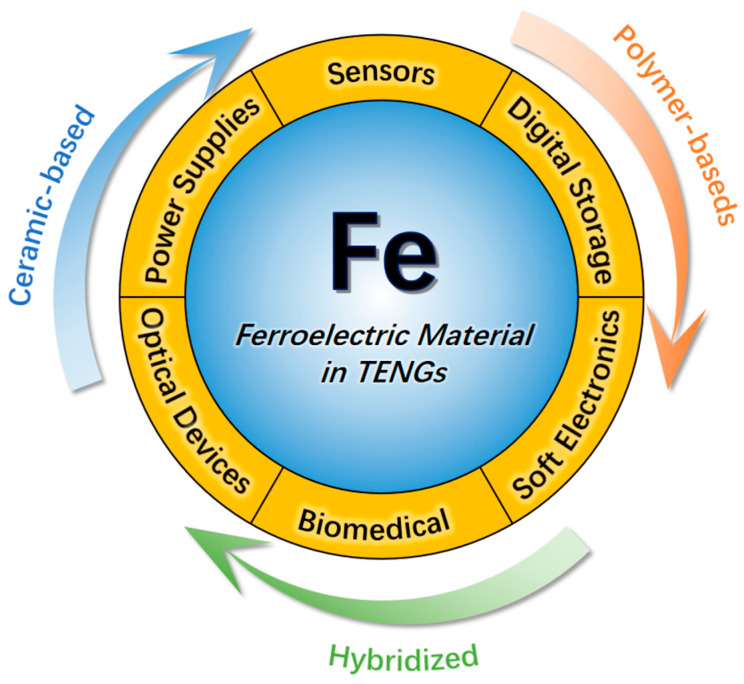
Progress of ferroelectric materials in TENGs.

The capacity to conduct reversible electrodeposition in the vicinity of an exterior powered by an electric field distinguishes a class of materials as possessing ferroelectric properties. The slightest hint of a field of electricity may trigger ionic deflections in the material structure, enabling the generation of ferroelectricity. This ferroelectricity can be reversed, and it imparts certain electrical performance characteristics to ferroelectric materials [26]. Ferroelectric materials are promising friction layer materials in TENGs [27]. This is because ferroelectric materials, with the characteristic of reversible electrode polarization under external mechanical force or pressure [28], boost the energy conversion efficiency of TENGs [29,30]. For instance, ferroelectric materials demonstrate exceptional dielectric characteristics and stability, which enables dependable and durable energy harvesting over extended periods [31,32]. Moreover, the piezoelectric properties of ferroelectric materials allow TENGs to effectively function under different strain settings [33,34] with high efficiency and stability. Furthermore, this application may endow the TENG-based electronics with versatility in digital storage, tactile sensing, and optical electronics, thus establishing a strong basis for future intelligent and adaptable technology applications (Figure 1) [35]. 

This review presents a thorough examination of the latest advancements in TENGs utilizing ferroelectric materials. It initially elucidates the operational principles of TENGs alongside the fundamental characteristics of ferroelectric materials. Subsequently, it delves into the structural design and material optimization of TENGs, with a particular emphasis on those utilizing ferroelectric materials. Additionally, it introduces novel methodologies aimed at enhancing the intelligence and multifunctionality of TENGs devices. Finally, this review offers a comprehensive synthesis of the future developmental trajectories and potential avenues for TENGs based on ferroelectric materials, thus furnishing a lucid and exhaustive perspective for forthcoming research endeavors.

## 2. Fundamentals of Ferroelectric Materials

### 2.1. Development History and Basic Properties of Ferroelectric Materials

Early in the 20th century, ferroelectricity was first discovered. The phenomena was first observed in 1921 by American scientist Joseph Valasek Roper, who was investigating the ferroelectric crystal barium titanate (BaTiO_3_) [36,37]. As time went on, more and more studies were conducted on the physical characteristics and uses of ferroelectric materials. Ferroelectric materials began to be used in industry with the advancement of technology and a deeper understanding of their properties [38]. Ferroelectric ceramic materials emerged as a significant component in capacitors and piezoelectric components [39]. Ferroelectric materials are also being used in sensors [40], medical devices [41], acoustic devices [42], and other industry sectors [43]. New varieties of ferroelectric materials, including multiferroelectric materials [44] and ferroelectric thin films [45], were found during the start of the twenty-first century. The physical properties of these materials are richer and more complicated, opening up new applications for ferroelectric materials [46,47]. In the last few decades, the field of nanotechnology has led to a growing interest in studying ferroelectric materials at the nanoscale and exploring different applications for them [48,49]. Investigation into the possibility of ferroelectric nanomaterials in energy harvesting [50], nanogenerators [51], and nanoelectronic devices [52] by researchers has opened up new avenues for ferroelectric material development in the future.

**Figure 2:**
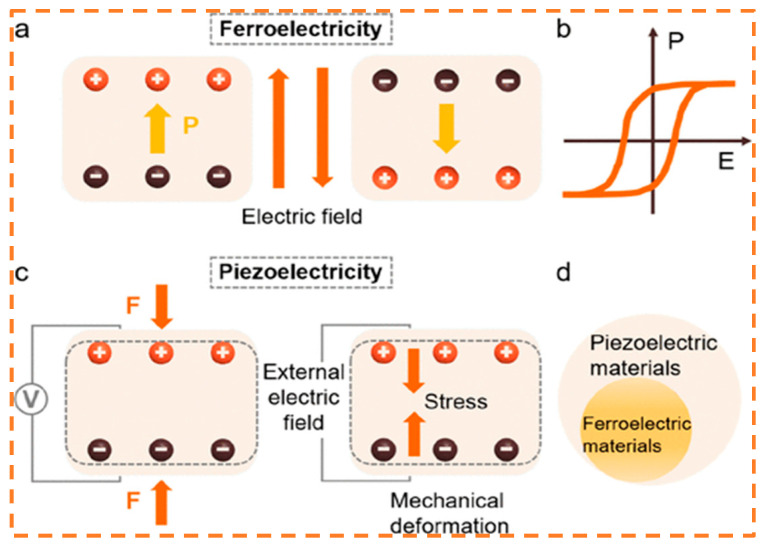
Illustrations depicting the operational mechanics of ferroelectric materials. (**a**) Ferrous electricity: the orientation of the polarization can be altered by a field of electricity that is external. (**b**) Diagram illustrating the ferroelectric reversal looping. (**c**) Piezoelectricity is utilized to convert physical power into electrical current. (**d**) One subclass of piezoelectric materials is ferroelectric materials. Reprinted with permission from Ref. [56] Copyright 2023, American chemical society.

The ferroelectric effect is one of the distinctive electrical characteristics of ferroelectric materials. The proposed ferroelectric effect refers to the occurrence of spontaneous electrode polarization in a material when subjected to an electric field that comes from the outside [53]. This is a result of the fact that the eccentric displacement of the positive and negative ions within the ferroelectric material produces an electric dipole moment even in the absence of an electric field from outside. Furthermore, a field of electricity being applied can alter the orientation of spontaneous polarization, producing an electric hysteresis return line [54], as shown in Figure 2a,b. In contrast, non-ferroelectric materials generally lack this characteristic. Within TENGs, the frictional interaction or external mechanical manipulation of two materials leads to charge separation. The incorporation of ferroelectric materials can amplify this phenomenon of charge separation, consequently enhancing the output efficiency of the TENG. In Figure 2d, ferroelectric materials exhibit the piezoelectric effect, which occurs when any outside force causes opposing and equated charges to form on two corresponding faces of the material due to electrodepolarization [28]. The inverse piezoelectric effect, as represented in Figure 2c, pertains to the deformation and mechanical stress of a material resulting from an electrical field being applied to it [46,55]. In the context of TENGs, ferroelectric materials fulfill a crucial role as the friction layer. Their inherent piezoelectric properties allow them to polarize under external forces, thereby inducing charge separation and facilitating electric current generation. This piezoelectric phenomenon underpins TENG’s efficient conversion of mechanical energy into electrical energy, serving as a fundamental pillar for its high-performance operation.

### 2.2. Classification of Ferroelectric Materials

**Figure 3:**
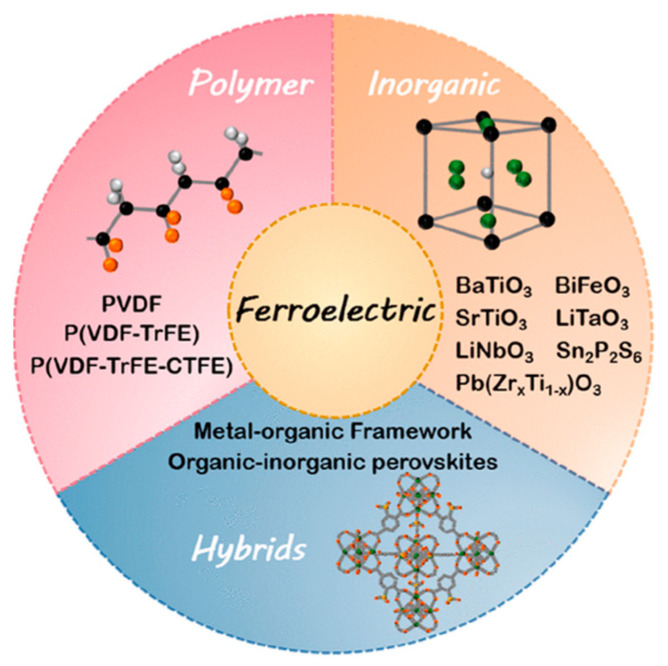
Classification of ferroelectric materials. Reprinted with permission from Ref. [56] Copyright 2023, American chemical society.

As shown in Figure 3, ferroelectric materials are capable of being categorized into three key categories according to their composition: ceramic-based (BaTiO_3_, BiFeO_3_, and Pb(Zr_x_Ti_1−x_)O_3_), polymer-based (polyvinylidene fluoride (PVDF)), and hybridized (compounds containing both inorganic and organic components chalcogenides and compounds with metal atoms bonded to organic backbones) materials [57].

#### 2.2.1. Polymer-Based Ferroelectric Materials

Polymer-based ferroelectric materials primarily encompass high-polymer materials exhibiting ferroelectric properties, exemplified by commonly employed PVDF and P(VDF-TrFE) [58]. Despite typically displaying lower dielectric constants and piezoelectric coefficients, their exceptional flexibility, cost-effectiveness, and lightweight nature render them immensely valuable in the realm of flexible electronics [59]. Within the domain of TENGs, polymer-based ferroelectric materials, owing to their exceptional mechanical flexibility and affordability, prove conducive to the fabrication of flexible and wearable devices. Their capacity to readily generate charges through external mechanical stimuli renders them advantageous for energy harvesting and sensor applications [60,61,62].

#### 2.2.2. Ceramic-Based Ferroelectric Materials

Ceramic-based ferroelectric materials predominantly comprise inorganic crystals or polycrystals, exemplified by barium titanate (BaTiO_3_), lead zirconate titanate (PZT), and bismuth ferrite (BiFeO_3_). The materials in question generally demonstrate high dielectric constants, piezoelectric coefficients, and ferroelectric conversion efficiency. Moreover, they demonstrate structural stability, resistance to high temperatures, and suitability for high-performance applications [63]. Within the realm of TENGs, ceramic-based ferroelectric materials, owing to their high piezoelectric coefficients and ferroelectric properties, can efficiently generate a substantial charge with minimal deformations, thereby augmenting energy conversion efficiency [31,64].

#### 2.2.3. Hybrid Ferroelectric Materials

Hybrid ferroelectric materials comprise two components: organic and inorganic, with the objective of amalgamating the benefits of both. These materials may manifest as either possessing an organic–inorganic perovskite structure or as composite materials blending inorganic ferroelectric particles with polymer matrices, such as the PVDF and inorganic nanoparticles composites [37,65]. They embody both the processing convenience and flexibility inherent in organic materials, along with the high-performance characteristic of inorganic counterparts [66]. Within the applications of TENGs, hybrid ferroelectric materials, through the synergy of polymer flexibility and the efficient energy conversion properties of ceramics, can optimize performance across diverse applications [34].

### 2.3. The Significance of Ferroelectric Materials in the Development of TENGs

Recent research indicates that ferroelectric materials, owing to their innate spontaneous polarization and high dielectric constant, hold promising prospects across various electronic device domains. Numerous studies further suggest that ferroelectric materials exhibit significant potential in TENGs. The output functions for the open-circuit voltage and short-circuit current of TENGS are represented by the following equations [67]:

(1) $$V=\sigma_{1}(z,t)\left[\frac{d_{1}}{\varepsilon_{1}}+\frac{d_{2}}{\varepsilon_{2}}\right]+\frac{z\left[\sigma_{1}(z,t)-\sigma_{c}\right]}{\varepsilon_{0}}$$

(2) $$RA\frac{d\sigma_{1}(z,t)}{dt}=z\frac{\sigma_{c}}{\varepsilon_{0}}-\sigma_{1}(z,t)\left[\frac{d_{1}}{\varepsilon_{1}}+\frac{d_{2}}{\varepsilon_{2}}+\frac{z}{\varepsilon_{0}}\right]$$

In the above equation, z is a function of time, ε_1_ and ε_2_ are the relative dielectric constants of the two contacting materials, d_1_ and d_2_ are the displacements of the two contacting materials, σ_1_ (z, t) represents the accumulation of free electrons in the electrode, σ_c_ is the surface charge density of the frictional electric charge, and A is the contact area size. Thus, under the same motion pattern and condition, the polarization of ferroelectric materials can control surface potential, increase surface charge density, and *potentiate difference*.

**Figure 4:**
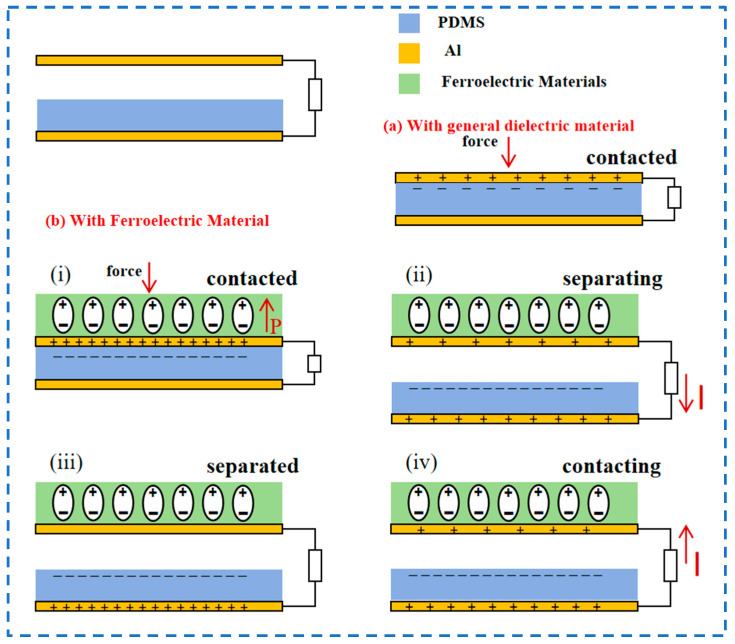
Comparison of working process of TENG based on ferroelectric materials and common dielectric materials.

The operation principle of TENGs relies on friction and charge transfer between materials. In TENGs that do not incorporate ferroelectric materials, friction materials such as PTFE, cellulose, PVDF, PDMS, etc., are commonly utilized. Friction between these materials induces the transfer of electrons and charge separation, resulting in an electrostatic effect [16,17,18,19,20,21]. However, since these materials primarily exploit the contact charging phenomenon, they are constrained by surface effects, hindering efficient energy conversion. In contrast, TENGs employing ferroelectric materials harness both the ferroelectric effect and piezoelectric effect, with their operational principle involving more pronounced polarization and charge separation within the material [68]. When two materials are rubbed together in a TENG, electron movement is stimulated, leading to surface charge separation on the materials [69]. This friction-induced electron transfer between the materials generates charge states characterized by positive and negative charge separation. In the case of ferroelectric materials, their inherent polarization further facilitates electron movement and separation. Ferroelectricity renders the surface charge of the material more responsive to polarization-induced changes, thereby enhancing electron mobility and separation efficiency [70,71,72] (Figure 4). Consequently, the integration of ferroelectric materials in TENGs can augment the charge output and energy conversion efficiency of the device.

**Figure 5:**
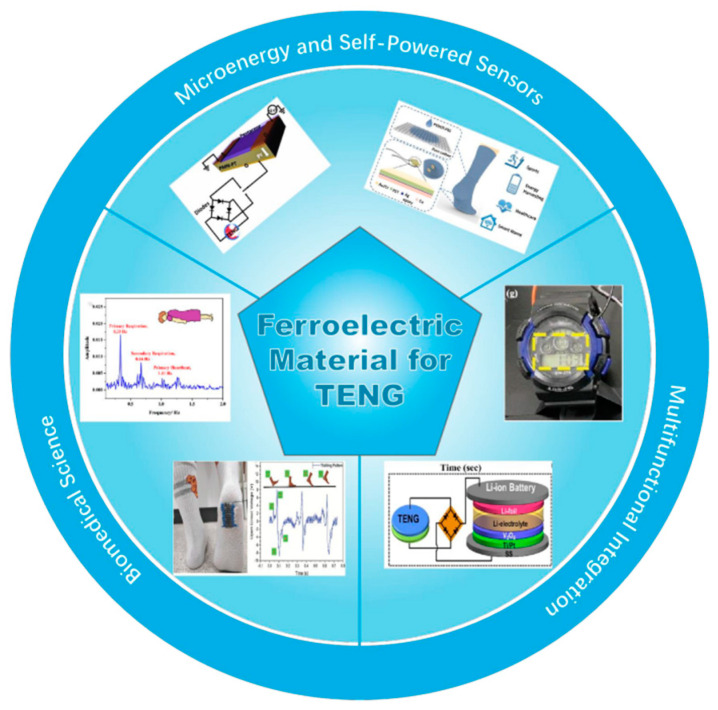
Progress of TENG for ferroelectric materials.

In summary, integrating ferroelectric materials into TENGs represents a significant advancement in energy conversion technology. Unlike traditional TENGs, which rely solely on friction-induced charge transfer, TENGs based on ferroelectric materials utilize the ferroelectric effect and piezoelectric effect to enhance polarization and charge separation within the material. Additionally, the inherent polarization of ferroelectric materials further enhances the efficiency of electron migration and separation, thereby increasing the device’s charge output and overall energy conversion efficiency. This innovative approach highlights the crucial significance of ferroelectric materials in advancing the performance and applicability of TENG technology in various energy harvesting applications [73,74] (Figure 5).

## 3. Basic Principles of TENGs

**Figure 6:**
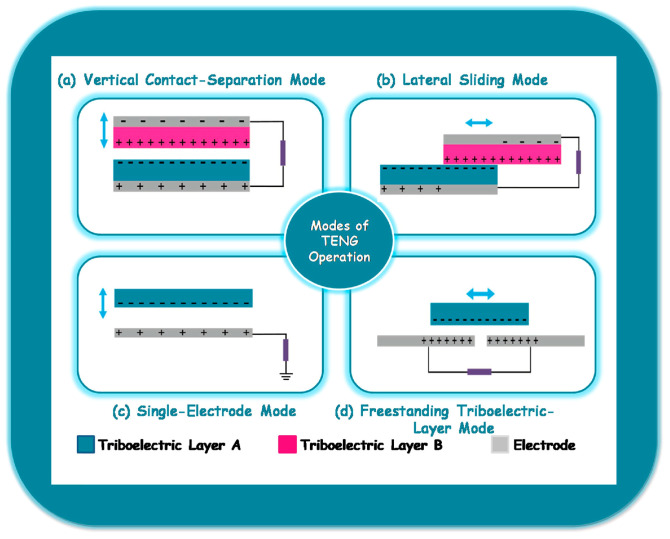
Diagrammatic representations depicting the operational mechanism of TENGs. (**a**) The VCS mode refers to vertical contact separation. (**b**) The LS mode refers to lateral sliding. (**c**) The SE mode refers to the operation of a single electrode. (**d**) The FT mode refers to the operation of the freestanding triboelectric-layer phase. Reprinted with permission from Ref. [82] Copyright 2020 Elsevier Ltd.

Originally developed for effectively changing movement energy into electric energy using electrostatic coupling and the friction electric effect [75], TENGs are a new class of energy harvesting device that can now be used as a sensor [76] and a power source [77] to create self-powered systems for a wide variety of applications. The triboelectric effect describes the emergence of electric charges when two dissimilar materials with varying surface electron affinities come into contact [78]. Friction causes the surfaces of the two materials to separate, producing different electrical potentials [79]. When an external load is imposed, electrons go through one side of the electrode to another aiming to balance the possible disparity; this process is known as electrostatic induction. TENGs utilize electrostatic induction and friction energization to transform kinetic energy into useful energy for electricity [80]. Besides that, TENGs operate based on four distinct modes: the vertical contact-separation setting, the lateral-sliding setting, the single-electrode setting, and the unsupported triboelectric-layer setting [81], as illustrated in Figure 6.

### 3.1. The Vertical Contact-Separation (CS) Mode

Figure 6a illustrates the common use of two different materials as the friction layer in a vertical contact-separation triboelectric nanogenerator (VCS-TENG). This device consists of two movable electrodes installed at the very top as well as bottom, which move vertically with respect to each other. When both layers of friction come into contact, charges of identical magnitude but opposing polarity are generated on the surfaces in contact, as a result of electron affinity. The two friction layer materials are separated when an external force acts upon them [82]. Electrostatic induction causes the movement of liberated electrons through electrodes, resulting in the creation of a reversed potential. This reversed potential generates an electrical flow in the circuit outside the electrodes, facilitating the conversion of energy. This is the most basic mode of operation for TENGs. Because of its vertical contact and separation working mode, it improves the efficiency of energy conversion by efficiently converting frictional energy into electric energy. Additionally, the functioning mode of VCS-TENG is highly robust and reliable due to its reduced susceptibility to external environmental influences, which sets it apart from some classic friction generators [81,83,84,85].

### 3.2. The Lateral-Sliding (LS) Mode

The lateral-sliding triboelectric nanogenerator (LS-TENG) and the vertical contact-separation triboelectric nanogenerator (VCS-TENG) work based on the same principle, which utilizes the electrostatic friction effect and the principle of electrostatic induction to switch movement energy into the electricity. The difference lies in the lateral-sliding method of operation that LS-TENGs utilize in comparison. Typically, the electrodes glide laterally across a level plane, as shown in Figure 6b. While slipping, they use electrostatic friction to create charges, resulting in surfaces with varying charges. A potential difference between the electrodes is produced by this relative sliding motion, and this difference in potential produces a current for energy conversion [86]. Owing to the various operating modes, VCS-TENG is better suited for situations requiring vertical motion, like some mechanical vibration applications, whereas LS-TENG is better suited for situations requiring lateral sliding, like wearable technology, flexible electronic products, and other fields [81,83,84,85].

### 3.3. The Single-Electrode (SE) Mode

The two aforementioned motion types differ from the single-electrode mode. In VCS-TENG, electrodes typically move in a vertical direction, generating charge differences through the process of contact and separation; in LS-TENG, the electrodes typically move in a lateral-sliding mode, using electrostatic friction during the sliding process to generate charges; and in SE-TENG, only a single electrode rubs against the external material, generating a change in the distribution of electrostatic charges, which generates a current that realizes energy conversion, as shown in Figure 6c. In contrast to many intricate multi-electrode mode friction generators, SE-TENG has a comparatively straightforward structure and minimal preparation costs, requiring only one electrode to achieve energy conversion [81,83,84,85].

### 3.4. The Unsupported Triboelectric-Layer (FT) Mode

The freestanding triboelectric-layer mode, which uses a friction substance and two electrodes as its usual components, works as seen in Figure 6d. Electrostatic induction results in the attraction of electrons towards a certain electrode when a positively charged friction electric substance is in close contact to that electrode that is used. When the friction electric substance is in close contact to it, electrons move from the initial electrode to the other electrode. An external load receives the current output produced by the electrodes’ relative motion back and forth in order to convert energy. In this mode, there is no electrostatic shielding effect, which improves output performance [87]. Moreover, due to its user-friendly nature, versatility, and exceptional effectiveness, FT-TENG may be applied to many friction surfaces, encompassing diverse materials and forms. Additionally, it is well-suited for utilization in autonomous sensor networks and pliable gadgets with electronics [81,83,84,85].

## 4. Regulation Strategies for Enhancing the Performance of TENGs Utilizing Ferroelectric Materials

### 4.1. Surface Engineering

Surface engineering is a multidisciplinary field dedicated to enhancing and controlling material surface properties through various technological methods to meet specific application needs. Initially focused on metal surface treatments like electroplating, heat treatment, and coating technologies to improve corrosion, hardness, and wear resistance, the discipline has since expanded to encompass non-metallic materials such as ceramics, plastics, and composites. Its diverse applications span numerous industries including aerospace, automotive, biomedical, energy, electronics, and communications [88]. Within aerospace, surface engineering techniques bolster material fatigue, corrosion, and high-temperature oxidation resistance [89]. In biomedical contexts, surface modification enhances implant biocompatibility and fosters cell growth [90], while in energy applications, optimizing battery electrode surfaces significantly enhances performance and longevity [91]. Additionally, surface engineering approaches are essential in enhancing energy utilization and maintaining stability in devices, as demonstrated in TENG research [92,93].

Ferroelectric materials are instrumental in augmenting the performance of TENGs, particularly through the utilization of surface engineering methodologies such as surface coatings [93], doping [94], and patterning [95]. Integration of these surface engineering techniques yields notable enhancements in the resulting voltage, current, and power density of TENGs (Table 1), thereby paving the way for novel opportunities in their practical applications and performance optimization.

#### 4.1.1. Surface Modification and Doping

**Figure 7:**
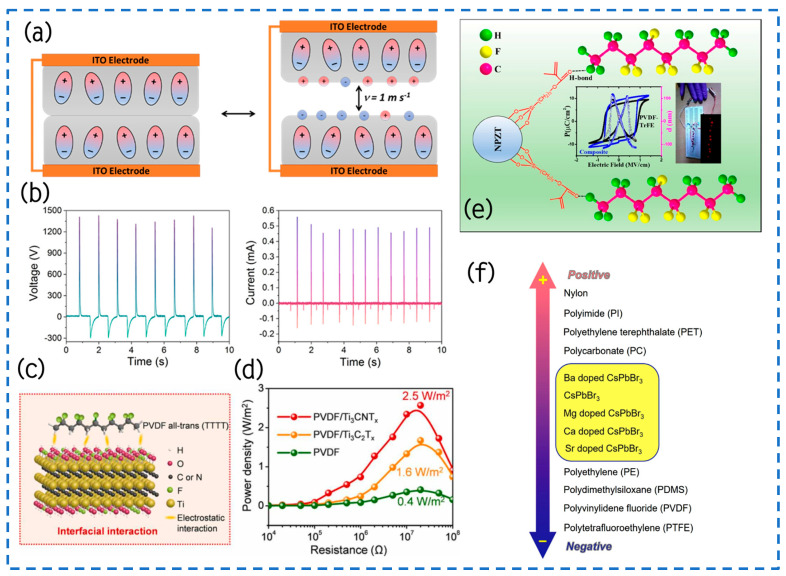
(**a**) Diagram depicting the TENG setup and the production of theoretical charges on the surface in reaction to the piezoelectric dipole. (**b**) Measurement of Isc and Voc. Reprinted with permission from Ref. [96] Copyright 2023 American Chemical Society. (**c**) A diagram is presented to illustrate the interface interaction among MXene nanosheets with PVDF strands. (**d**) The immediate electricity density of TENGs fabricated with PVDF/0.4 Ti_3_CNT_x_, PVDF/0.4Ti_3_C_2_T_x_, and PVDF was evaluated. Reprinted with permission from Ref. [92] Copyright 2021 Elsevier Ltd. (**e**) The graphical representation depicts the communication that occurs between the PVDF-TrFE matrices and TMSPM-modified NPZT nanoparticles in the PVDF-TrFE matrix. Reprinted with permission from Ref. [94] Copyright 2019 American Chemical Society. (**f**) The qualitative frictional electrification polarity of pristine CsPbBr_3_ and CsPb_1−x_M_x_Br_3_ perovskite materials was showcased. Reprinted with permission from Ref. [97] Copyright 2020 Elsevier Ltd.

Recently, Šutka et al. showcased notable advancements in the energy conversion efficiency of TENGs by integrating ferroelectric materials like β-PVDF and ferroelectric ceramic particles at the TENG contact interface (Figure 7a) [96]. Importantly, this addition not only bolstered the electrostatic induction strength but also augmented the efficiency of mechanical-to-electrical energy conversion, especially evident when the polarization states of these ferroelectric elements displayed depolarization characteristics. Notably, through techniques like pressure folding and reverse polarization treatment, Šutka et al. successfully synthesized polyvinylidene fluoride (PVDF) with up to 88% β-phase, laying a robust foundation for enhancing performance of TENGs significantly. Experimental results showcased the capabilities of a depolarized β-PVDF TENG with a mere 5 cm^2^ area, yielding open-circuit voltages of upwards of 1350 V and short-circuit currents of 0.5 mA under the contact-separation mode (Figure 7b). Remarkably, the device demonstrated an impressive highest power density of 24 W/m^2^, ranking among the highest reported performance metrics for PVDF-based TENGs. This exceptional performance stems from the well-ordered arrangement of ferroelectric dipoles within PVDF polymers, boasting an unusually high 88% piezoelectric β-phase content. This study highlights the crucial significance of ferroelectric materials in improving the effectiveness of TENGs and also introduces an entirely novel path toward the development of efficient energy conversion devices.

Recently, the research team lead by Song et al. successfully modified and increased the production power of TENGs by a dielectric modulation technique produced by interface interactions, thus opening up a new optimization pathway (Figure 7c) [92]. Specifically, they carefully selected two types of two-dimensional transition metal carbides, namely titanium-based carbides (Ti_3_C_2_T_x_) and titanium-based carbonitrides (Ti_3_CNT_x_) MXene, and incorporated them into polyvinylidene fluoride (PVDF) ferroelectric polymers to attain optimal control over dielectric performance modulating. Experimental results show that the optimized PVDF/Ti_3_CNT_x_-based TENG outperforms a device based on pristine PVDF (0.4 W/m^2^) and PVDF/Ti_3_C_2_T_x_ (1.6 W/m^2^). When the matching impedance is set to 20 MΩ, the TENG device based on PVDF/0.4 Ti_3_CNT_x_ film accomplishes an output power density of up to 2.5 W/m^2^ and maintains excellent stability during repeated contact-separation cycles for up to 100,000 times (Figure 7d). This efficient electromechanical energy conversion capability enables the TENG device to power a set of parallel commercial red, white, and blue LED lights, and even an electronic wristwatch, demonstrating its significant potential in distributed energy harvesting and utilization. By introducing strong interface interactions to achieve effective dielectric modulation, this work not only broadens the new pathways for the material design of TENGs and performance optimization but also provides a strong scientific basis and technical guidance for the advancement of next-generation energy-capturing machines with superior performance.

Gupta et al.’s research team proposed an innovative solution to address the brittleness inherent in traditional ceramic-based piezoelectric materials and the relatively low electrical output of polymer-based alternatives [94]. They devised a composite material comprising a piezoelectric polymer matrix (PVDF-TrFE) filled with nanosized particles of niobium-doped Pb(Zr,Ti)O_3_, aiming to achieve the desired flexibility and increased electrical output. By modifying the nanoparticles with trimethoxysilylpropyl methacrylate (TMSPM), they effectively improved the connection that links filler with the matrix, making it easier for local dipole–dipole interactions to occur (Figure 7e). At a frequency of 100 Hz, the residual polarization of this composite material rose to 9.1 µC/cm^2^, with a longitudinal piezoelectric coefficient of 101 pm/V. Demonstrating the capabilities of this composite material, the research team exhibited a piezoelectric nanogenerator (PENG) capable of generating a 10 V output through mechanical bending. Compared to conventional polymer PVDF-TrFE film devices, this composite device showcased a remarkable over 200% increase in output. Moreover, these composite materials found application in TENGs, efficiently supplying energy to 10 industrial red lightbulbs. The combination of a piezoelectric nanogenerator and TENGs in this device shows promise as an environmentally sound and dependable power supply for wireless sensor nodes and wearable medical gadgets.

Wang et al.’s team utilized a vertical contacted-separation TENG device for a comprehensive study on the frictional charge affinity and polarity of all-inorganic cesium lead tribromide (CsPbBr_3_) and alkaline earth ion-doped CsPb_1−x_M_x_Br_3_ (M = Ca^2+^, Ba^2+^, Mg^2+^, Sr^2+^, x = 0–1) perovskite materials [97]. Through the assessment of electrical output parameters (Voc, Isc, Qsc, and FOM_p_) and work function, they conducted a quantitative analysis of the perovskite materials’ frictional electrification series. Their findings indicated a polarity sequence from negative to positive as CsPb_0.93_Sr_0.07_Br_3_ < CsPb_0.97_Ca_0.03_Br_3_ < CsPb_0.99_Mg_0.01_Br_3_ < CsPb_0.91_Ba_0.09_Br_3_, showcasing the methodology’s relevance and practicality for exploring the intrinsic frictional charge affinity of novel materials (Figure 7f). Moreover, by integrating the triboelectric and photovoltaic effects, reverse voltage signals and a thousandfold increase in current signals were observed under illumination, highlighting the crucial impact of photo-induced charges on augmenting frictional charges and modifying the charging polarity of TENGs. This research not only expands the frictional electrification series for unfamiliar materials but also establishes a basis for advancing high-performance optoelectronic detectors.

#### 4.1.2. Surface Coating

**Figure 8:**
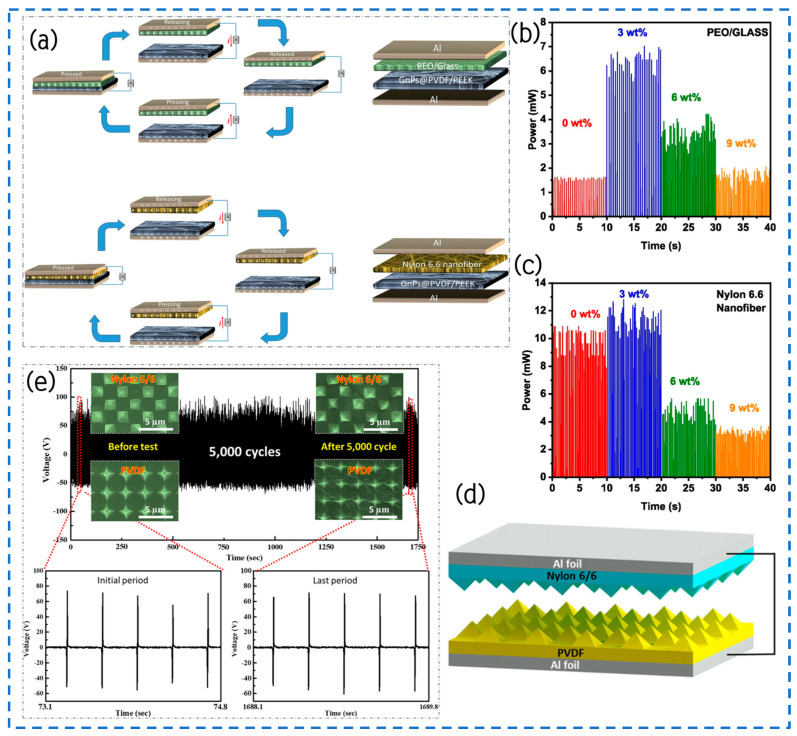
(**a**) The TENGs operate based on the operating concept of GnPs@PVDF/PEEK and Nylon 6.6 nanofibers and GnPs@PVDF/PEEK and PEO/Glass. (**b**) The immediate power results at 1.1 MΩ change based on the ratio of GnPs additive in the PEO Glass veil and GnPs@PVDF/PEEK TENG. (**c**) The immediate power results at 1.1 MΩ of the Nylon 6.6 nanofibers and GnPs@PVDF/PEEK TENG change based on the ratio of GnPs additive. Reprinted with permission from Ref. [93] Copyright 2022 Elsevier Ltd. (**d**) A simplified diagram depicting the configuration of a TENG device utilizing a patterned polymer film is showcased. (**e**) Conduction of 5000 cycles of strength and rigidity testing on the TENG utilizing pyramid-patterned nylon and PVDF sheets. Reprinted with permission from Ref. [95] Copyright 2021 Wiley-VCH GmbH.

Okbaz employed GnPs@PVDF/PEEK as the negative friction electrode layer, while utilizing Nylon 6.6 nanofibers and a PEO/Glass sheet as the positive friction electrode layer to fabricate a TENG that operates with the vertical contact-separation mechanism (Figure 8a) [93]. In this investigation, the PEEK surface was coated with a PVDF solution to fully exploit its excellent friction-negative and piezoelectric properties. This coating reduced the surface friction coefficient, minimizing energy dissipation during friction, thereby enhancing the wear resistance and prolonging the service life of the friction components. Furthermore, the incorporation of GnPs into PVDF resulted in a variable increase in the material’s dielectric characteristic and an enhancement of the surface roughness of the PVDF/PEEK synthetic materials. Experimental results demonstrated optimal output power for various GnPs@PVDF/PEEK compositions combined with Nylon 6.6 nanofibers or PEO/Glass film when the load resistance was set at 1.1 MΩ. Notably, for 3 wt.% GnPs@PVDF/PEEK and PEO/Glass sheet and 3 wt.% GnPs@PVDF/PEEK and Nylon 6.6 nanofibers, output powers of 6.46 mW and 11.8 mW were achieved (Figure 8b,c), respectively, corresponding to a power density of 4.04 W/m^2^ and 7.36 W/m^2^. Consequently, GnPs@PVDF/PEEK emerges as a promising TENG candidate material, boasting high performance, compactness, and simplicity, thereby providing an innovative power strategy for lightweight, practical, and self-sustaining technological gadgets.

In a recent study, Uddin et al.’s team developed an innovative coaxial yarn-based triboelectric nanogenerator (Y-TENG), showcasing an efficient coating method on flexible stainless-steel threads using a melt-coating technique to enhance the dielectric medium—PVDF [98]. The main benefit of this approach is that it allows for the direct application of dielectric materials onto metal-based electrodes, without the need for any additional processing procedures. The implementation of this direct-coating technology streamlines the manufacturing process to a great extent and effectively decreases expenses. Specifically, when a force of 700 N was applied and a frequency of 2.11 Hz was used, the mean peak open-circuit voltage (Voc) was measured at 3.82 V, with a maximum Voc of 16.1 V. The median peak short-circuit current (Isc) was 12.96 nA, reaching a maximum Isc of 53.1 nA. Furthermore, the trials also evaluated the power output efficiency of Y-TENG when subjected to a 300 N force at a frequency of 2.11 Hz, with a load impedance of 560 Ω, achieving an output power of 1.05 mW/cm^2^. By employing a bridge rectifier with full waves for turning the electricity output into a charging capacitor, the team demonstrated Y-TENG’s potential to power wearable electronic devices. In addition, the study team conducted tests on Y-TENG to demonstrate its ability to harness human mechanical energy. These tests involved replicating human movements including finger clapping, running, and finger bending. The results revealed that during finger clapping, running, and finger bending, the max Voc reached 14.2 V, 7.2 V, and 1.8 V, respectively, while the peak Isc was 222 nA, 12.45 nA, and 67.5 nA, respectively. In summary, Uddin et al.’s work not only offers an efficient and cost-effective manufacturing method for Y-TENG but also demonstrates its enormous application potential in the wearable energy field through extensive performance evaluations. This comprehensive study is of significant importance in driving rapid prototyping and scalability in the development of wearable power-generating smart textiles.

#### 4.1.3. Surface Patterning

The research team led by Choi has devised a strategy employing straightforward phase transition techniques to enhance the efficacy of polyvinylidene fluoride (PVDF), nylon, and other polymer films within TENGs (Figure 8d) [95]. This approach significantly amplifies the film’s surface area via a swift and uncomplicated phase transition film formation process. Morphological analysis confirms the seamless transfer of patterns from the substrate to the polymer film, devoid of any imperfections. Significantly, the phase transition and crystallization of nylon and PVDF stay unchanged, even when there is surface patterning. This is because the cooling rate is consistently controlled during the phase transition process. The utilization of pyramid-shaped PVDF and nylon films exhibits a 5.8-times increase in output power compared to flat films. Furthermore, even after undergoing 5000 cycles of testing, the output voltage of TENG devices equipped with patterned films remains steadfast, illustrating remarkable resilience and stability against repetitive compression and release cycles due to the natural flexibility of the polymer materials (Figure 8e). This technology, rooted in enhanced phase transition methodologies, not only offers simplicity and expediency in operation but also holds widespread applicability across diverse scenarios, including the fabrication of micro-patterned films.

**Figure 9:**
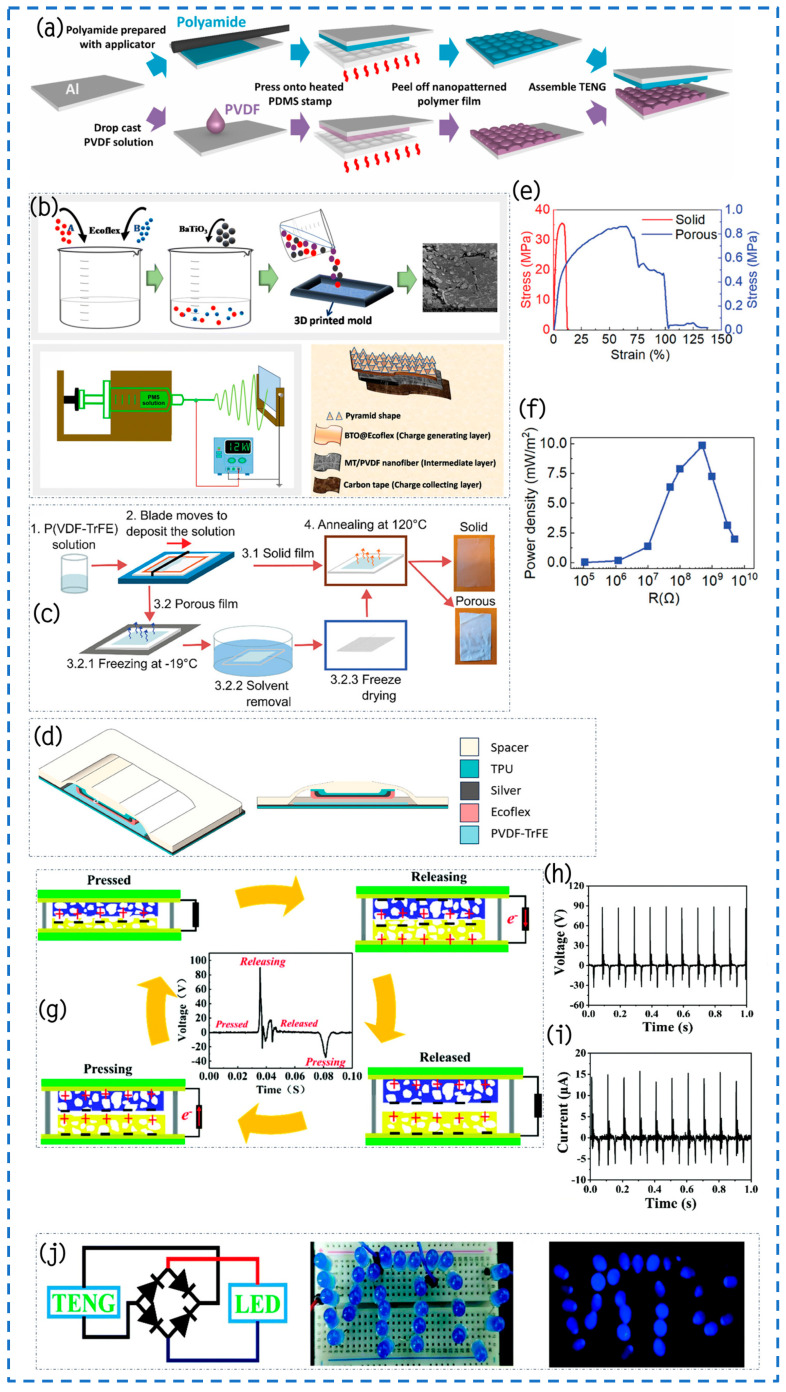
(**a**) A simplified illustration depicting the hot microcontact printing technique for manufacturing micro-patterned polyimide and PVDF surfaces is exhibited. Reprinted with permission from Ref. [99] Copyright 2021 Elsevier Ltd. (**b**) The manufacturing process of EPMTNG devices. Reprinted with permission from Ref. [100] Copyright 2024 Royal Society of Chemistry. (**c**) The process map illustrates the steps involved in preparing robust and P(VDF-TrFE) porous aerogel layers. (**d**) The structural schematic diagram of the TENG. (**e**) Understanding the mechanical properties of durable and P(VDF-TrFE) porous aerogel sheets requires analysis of their curves of stress and strain. (**f**) The average power density of the TENG. Reprinted with permission from Ref. [101] Copyright 2024 Wiley-VCH GmbH. (**g**) This paragraph depicts a schematic diagram that showcases the process of gathering and transforming energy using a β-phase PVDF aerogel TENG. (**h**) The Voc and (**i**) Isc values of the TENG. (**j**) Photographs showcasing the TENG connected to a series of 30 blue LEDs are presented. Reprinted with permission from Ref. [102] Copyright 2021 Royal Society of Chemistry.

Pradel et al. introduced a method leveraging simple geometric models. The approach utilizes nickel as a template and employs thermal microcontact printing technology to precisely pattern polyamide and polyvinylidene fluoride (PVDF), followed by tight integration with TENGs (Figure 9a) [99]. The study team conducted a thorough investigation into how the spacing of micro-patterns affects the efficiency of TENGs made from polyamide and PVDF. Experimental findings indicate that fine-tuning the spacing between pattern features not only leads to significant enhancements in output voltage and current compared to flat samples but also offers a fresh perspective on the controlling performance of TENGs. Additional numerical simulation studies are necessary in this field to enhance our comprehension of the interaction mechanisms, despite the initial research indicating a noteworthy enhancement in outputting of TENGs through the expansion of the material contact surface area. Particularly, in various TENG operating modes, such as the single-electrode mode or sliding mode, the development of more intricate models is necessary to capture these phenomena owing to the complex interactions involving surface disparities. Additionally, as the dimensions of patterned features approach the true nanometer scale, factors beyond surface enlargement may influence performance of the TENG. These factors encompass charge transfer, local field enhancement, and frictional characteristics of nanoscale contact surfaces, all of which play pivotal roles in the interactions between patterned surfaces. This not only opens avenues for future experimental design and theoretical analysis but also potentially unveils further optimization paths for TENG technology in energy harvesting and sensing applications.

Sarkar and his team have developed an innovative self-powered, skin-adhering flexible triboelectric nanogenerator (EPMTNG), constructed using micro-patterned BaTiO_3_@Ecoflex (EBTO) nanocomposites, two-dimensional layered MoTe_2_-reinforced PVDF nanofibers (PM5), and carbon strips (Figure 9b) [100]. The objective of surface modification of EBTO nanocomposites is to augment the surface areas and improve the surface potential. On the other hand, micro-patterned structures enhance the coarseness of the EBTO surface, hence expanding the region of effective contact between the EBTO layer and the hand. This modification encourages the formation of more friction charges when the EBTO layer contacts the hand, increasing the prospective contrast during the friction-electrification processes and greatly boosting the outcomes of the EPMTNG machine. When a steady force of 12 N was applied to the autonomous flexible apparatus, it produced an output voltage of up to 319 V and a current density of 9 mA/m^2^. At the same level of axial pressure, the device demonstrated an outstanding power density of 2.9 W/m^2^ when subjected to a load of 107 Ω. In addition, the study investigated how PM5 nanofibers affect the electrical performance of the device by conducting tests on its dielectric properties. The results showed that incorporating two-dimensional MoTe_2_ nanoparticles into the matrix of PVDF not only increased the amount of the β-phase, but also enhanced the effects of polarization by incorporating greater carriers of charge into the polymer matrix. As a result, the dielectric performance of the device was improved. This EPMTNG device exhibited great responsiveness (32.5 V/kPa) under pressures as low as 0.5 kPa, a characteristic highly applicable in tracking and wirelessly transmitting various human physiological signals to receiving devices such as laptops. Thus, this autonomous and adaptable technology has enormous possibilities for extensive utilization in the sectors of wellness tracking and soft automation.

#### 4.1.4. Porous Structure

Lozano Montero and colleagues introduced a novel study methodology that combines cryogenic molding, freeze-dried, and printed technologies. The researchers used porous aerogels made of P(VDF-TrFE) as substrates to create a completely manufactured TENG [101], as shown in Figure 9c,d. Additionally, they performed a methodical examination of how porous architectures affect the mechanical, magnetic, and triboelectric capabilities in contrast to strong P(VDF-TrFE) films. The outcomes demonstrated a substantial improvement in the tensile properties of P(VDF-TrFE) porous aerogels, with a rise from 7.7% in the solid phase to 66.4% in the porous state achieved through structural alterations (Figure 9e). The improvement resulted in a 66% rise in the voltage produced and an additional 48% in the generation of charge in non-polar P(VDF-TrFE) porous aerogel material films as contrasted to their solid equivalents. In addition, a TENG that utilizes stretchy materials and is completely printed demonstrated an average power output of 9.9 mW/m^2^ (Figure 9f) and a maximum power output of 62.8 mW/m^2^ during 100 separation cycles at a frequency of 0.75 Hz. Overall, porous aerogel films made of P(VDF-TrFE) exhibit exceptional tensile strength and triboelectric characteristics, indicating potential use in adaptable and elastic energy-capturing products.

Wang and colleagues utilized simple solvent replacement and freeze-drying techniques to fabricate β-phase porous PVDF aerogels, showcasing enhanced mechanical properties and superior triboelectric performance (Figure 9g) [102]. Consequently, this improvement greatly increased the triboelectric power of the generated TENGs. Operating at an input frequency of 10 Hz and subjected to a 16 N (0.08 MPa) cyclic pressure, the TENG produced an Voc of 90 V, a Isc of 15 mA (Figure 9h,i), and a computed power density of 6.15 W/m^2^. This development led to a threefold augmentation in voltage, an 8.2-fold surge in current, and an 8.2-fold enhancement in power density when compared to dense PVDF membranes. The TENG, utilizing β-phase porous PVDF aerogels, illuminated 30 blue LEDs (Figure 9j). Furthermore, the synthetically generated TENG demonstrated promise to be a remarkably responsive motion sensor that operates without external power. Non-crosslinked PVDF aerogels show great potential for exceptional TENGs and self-sufficient detectors, providing invaluable knowledge into the use of porous non-crosslinked polymer aerosols in the realm of energy collection.

**Figure 10:**
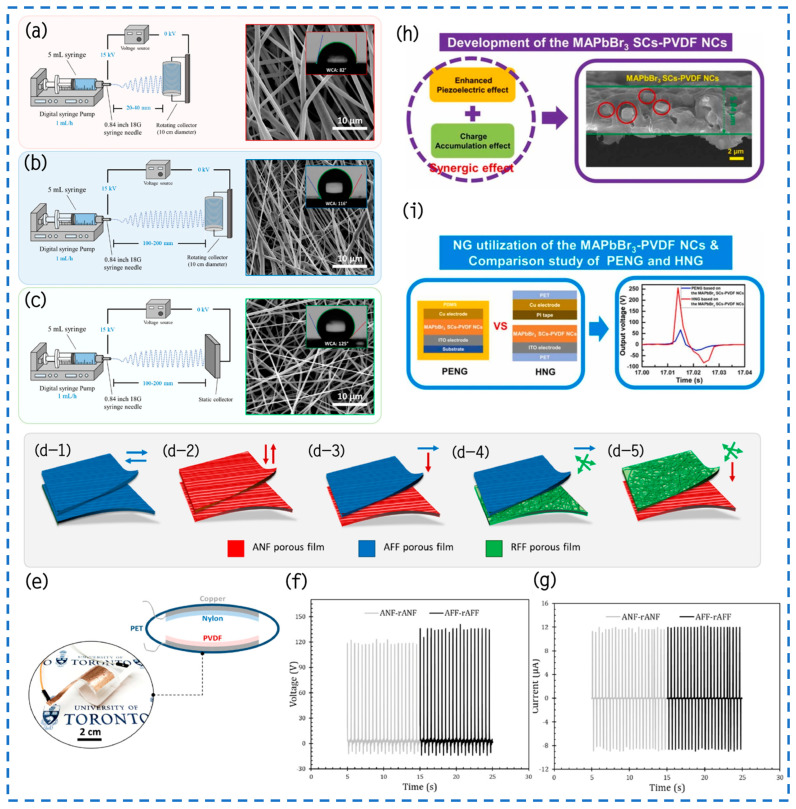
(**a**) This study examines the preparation method, WCA, and SEM comparison of electrically spun specimens with multiple arrangements of ANF, (**b**) AFF, and (**c**) RFF. (**d**) This paragraph examines the arrangement of electrically spun PVDF in several orientations and directions of its dipole: (**d-1**) AFF-rAFF, (**d-2**) ANF-rANF, (**d-3**) AFF-ANF, (**d-4**) AFF-RFF, and (**d-5**) ANF-RFF film. (**e**) Schematic diagram of curved TENG configuration. (**f**) Output voltage and (**g**) output current of multi-layer electrospun mats. Reprinted with permission from Ref. [103] Copyright 2023 Published by Elsevier Ltd. (**h**) The paragraph showcases the synergistic interplay between the heightened piezoelectric effect and increased charge accumulation. (**i**) An expanded depiction of the output voltage waveforms within a single cycle is presented for (PENG) and (HNG) configurations utilizing MAPbBr_3_ (25% wt.%)-PVDF NCs. Reprinted with permission from Ref. [104] Copyright 2022 Published by Elsevier Ltd.

Rastegardoost et al. have addressed the limitations of the previously developed electrospun mat for TENG devices, including its low dielectric performance, complex manufacturing process, and the need to incorporate additional nanoparticles to compensate for the presence of low dielectric air. To overcome these challenges, they have successfully conducted research and development on a porous electrospun mat made of PVDF. This mat exhibits significantly improved dielectric performance and features a novel configuration of dipoles [103]. Through meticulous manipulation of the electrospinning process parameters, they synthesized three distinct configurations of single-layer electrospun mats: ANF, AFF, and RFF (Figure 10a–c). This investigation revealed that strategic optimization of electrospinning parameters is crucial for the material’s structural, chemical, wettability, and mechanical enhancements. The discovery that the exact alignment of dipoles is crucial in increasing the dielectric constant of electrically spun felts is especially noteworthy. In the one-layer comparison examination, the AFF design had the highest dielectric constant, whereas the ANF and RFF configurations closely followed. The enhancement in dielectric performance became even more pronounced when these porous electrospun felts were stacked, maintaining specific dipole orientations. Notably, in a comparison of five different design configurations (Figure 10d), the dielectric constants of AFF-rAFF and ANF-rANF reached a value of 10, significantly surpassing that of the one-layer electrospun mat and rivaling the dielectric constant of the pristine, non-porous PVDF film. This breakthrough in material design, in conjunction with the use of nylon as a counter dielectric medium, facilitated the assembly of an arc-shaped TENG device (Figure 10e). The apparatus demonstrated an output voltage of over 130 V and a current of 12 μA (Figure 10f,g), which was significantly higher than that of the non-porous initial PVDF layer and single-layer electrospun mats. Crucially, the device maintained stable performance even after undergoing 10,000 testing cycles, underscoring its exceptional mechanical stability and durability. Further empirical tests, involving the integration of the TENG device into circuits bearing various external loads, indicated that an optimal external resistance of approximately 20 MΩ yields a maximum power density of 3.5 W/m^2^. Lastly, the energy storage capabilities of different capacitors were thoroughly examined, demonstrating their ability to achieve full charge under diverse conditions. This underscores the broad potential applications of the newly developed electrospun PVDF mat in the domains of energy harvesting and storage devices, heralding a significant advancement in the field.

In a recent groundbreaking study, Lee and his team utilized a simple and effective sol–gel method to synthesize a unique nanocomposite material comprising single-crystalline methylammonium lead tribromide (MAPbBr_3_) and polyvinylidene fluoride (PVDF) [104]. The pivotal technological advancement lies in the successful embedding of MAPbBr_3_ single crystals (SCs) in a highly uniform distribution within the porous structure of the PVDF matrix. In comparison to traditional pure PVDF membranes, the newly synthesized MAPbBr_3_ SCs-PVDF nanocomposite (NCs) membrane not only demonstrates an enhanced porous structure but also exhibits significant advantages in piezoelectric performance and charge accumulation effects (Figure 10h). Building upon the development of this nanocomposite material, the research team further conceptualized and fabricated a highly efficient piezoelectric/triboelectric hybrid nanogenerator (HNG), conducting extensive performance comparisons with a piezoelectric nanogenerator (PENG) exclusively. It is noteworthy that the HNG achieved an impressive output voltage of 256 V (Figure 10i), surpassing the output voltage of a similar nanocrystal-based PENG by a factor of 3.87. Moreover, the performance of the HNG in terms of power density was equally remarkable, with a value of 16.17 mA/cm^2^, approximately 200 times that of a similar NCs-based PENG. Through this uniform embedding technique, the research team not only surmounted the limitations of previous materials concerning mechanical and electrical performance but also introduced a novel strategy for designing and fabricating efficient HNGs based on high-performance halide perovskite single crystals integrated into porous PVDF matrices.

### 4.2. Micro- and Nanoscale Engineering

The evolution of micro–nano engineering can be traced back to the 1960s, marked by a series of groundbreaking milestones. Notably, Richard Feynman’s influential talk “There’s Plenty of Room at the Bottom” in 1959 is particularly noteworthy, as it introduced the concept of manipulating matter at the nanoscale for the first time. This perspective is widely acknowledged as a revolutionary revelation in the field of nanoscience and technology [105]. In addition, propelled by the emergence and advancement of scanning probe microscopy techniques, alongside the discovery of nanomaterials like carbon nanotubes, micro-nano engineering has entered a new phase of development. During this era, scientists have achieved precise manipulation of materials at the atomic and molecular levels, significantly broadening the scope of applications in various fields, ranging from semiconductor devices to biomedicine, energy storage and conversion, and information technology. Today, micro–nano engineering remains a dynamically evolving domain, not only fostering technological advancements but also offering novel solutions and technologies to address global energy, environmental, and health challenges [106].

Engineering ferroelectric materials at the micro- and nanoscale, including the preparation of nanowires, nanoarrays, and nanoparticles for applications, has become one of the key research directions in the field of materials science and nanotechnology [107,108,109]. Ferroelectric materials possess distinctive attributes of spontaneous polarization and the ability to alter their polarization orientation subjected to the effects of an outside electrical field [57]. This inherent trait renders TENGs based on ferroelectric materials highly promising for applications in information storage, sensors, and energy conversion across various domains [39,40,73]. By utilizing micro-nano engineering techniques, researchers are able to exert exact control over the characteristics of these materials, hence facilitating the creation of TENGs that are highly integrated, are multifunctional, and exhibit outstanding performance (Table 1).

**Figure 11:**
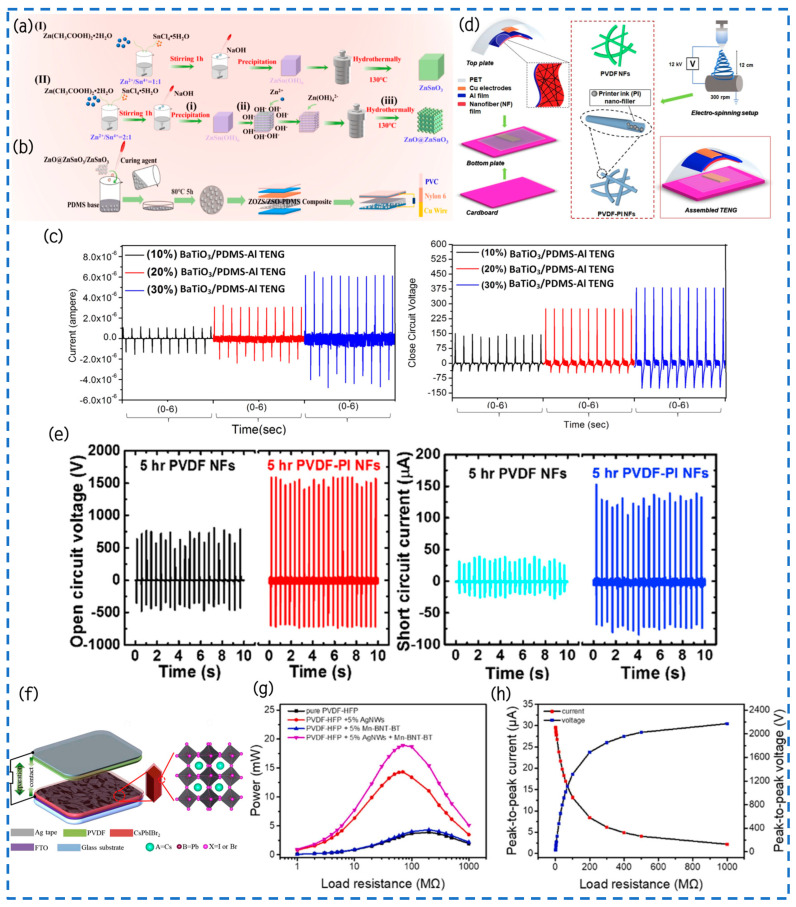
(**a**) A diagram depicting the process of preparing cubic ZnSnO_3_ and ZnO@ZnSnO_3_. (**b**) This schematic figure depicts the manufacturing process of the mixed film and TENG. Reprinted with permission from Ref. [27] Copyright 2023 American Chemical Society. (**c**) This figure demonstrates the output performance of the BaTiO_3_/PDMS-Al-based TENG. Reprinted with permission from Ref. [109] Copyright 2017 IOP Publishing Ltd. (**d**) This showcases the operational principle of the perovskite TENG. Reprinted with permission from Ref. [111] Copyright 2020 Elsevier Ltd. (**e**) The fabrication process involves the creation of TENGs utilizing PVDF-PI nanofibers and electro-spun PVDF nanofibers. (**f**) The Isc and Voc values of PVDF NFs and PVDF-PI NFs-based TENGs. Reprinted with permission from Ref. [107] Copyright 2020 Elsevier Ltd. (**g**) This diagram illustrates the power produced from several distinct TENGs. (**h**) This diagram depicts the efficiency of the output current and voltage of the TENG when utilizing a fiber mat made of 5% AgNWs + PVDF-HFP + 5% Mn-BNT-BT. Reprinted with permission from Ref. [108] Copyright 2022 Elsevier Ltd.

The hydrothermal method was employed by Jiao and their team to successfully synthesize ZOZS and ZSO nanoparticles [27]. According to Figure 11a, they directly grew semiconductors of ZnO on high-purity, self-polarized, lead-free ferroelectric ZnSnO_3_ nanoparticles. Moreover, Figure 11b elucidates the preparation procedure of ZSO/ZOZS-PDMS composite thin films and their associated TENG. This technological advancement facilitates the homogeneous dispersion of ZOZS nanoparticles within the ZSO/ZOZS/PDMS flexible composite membrane, effectively mitigating the aggregation of numerous ZSO nanomaterials dispersed in a PDMS mixture. Due to the combined effects of electric induction and piezoelectricity, the performance of this composite membrane is greatly improved. It demonstrates an open-circuit voltage of 218 V, a density of power of 24.625 μW/cm^2^, a charge transfer of 60 nC, and a short-circuit current of 14.2 μA. In comparison to TENGs based on pure PDMS, the output voltage, charge transfer, and current are amplified by 6.8 times, 10 times, and 9.7 times, respectively. This engineered TENG not only effectively illuminates 212 LED lights but also has the capability to charge electronic devices autonomously without necessitating additional chargers.

In this study, Ali et al. utilized a novel approach by blending tetragonal ferroelectric BaTiO_3_ nanocrystals with a polydimethylsiloxane (PDMS) matrix to fabricate a lead-free BaTiO_3_/PDMS-Al composite material for the production of working electrode films in plant-based TENGs [109]. With the goal of boosting the efficiency of the TENG, a post-polarization approach was implemented. By applying a positive potential of +10 kV, the dipole structures of tetragonal BaTiO_3_ nanocrystals were aligned, imparting a high positive polarity to the entire cured film surface throughout the process. According to the data in Figure 11c, our composite film exhibited the maximum electrical output signal at a current density of 0.3 μA/cm^2^ when the concentration of embedded barium titanate nanocrystals was 30 wt.%, reaching 375 V and 6 μA. By utilizing a load resistor of 100 MΩ, the power output was able to achieve 2.25 mW, which proved to be adequate for sequentially illuminating 100 LEDs. This work also proposes a possible method to improve the product performance of additional inorganic materials that include dipole structures by using the post-polarization methodology. The composite film of TENGs provides a broad spectrum of applications owing to its stability, adaptability, and biocompatibility. Furthermore, this research contributes to the exploration of novel TENG devices with enhanced output performance.

Kim and colleagues proposed a high-performance TENG and integrated it synergistically with a three-dimensional custom interface [110]. They initially selected aluminum foil and polyvinylidene fluoride-trifluoroethylene (PVDF-TrFE) nanofiber pads as positive and negative electrode materials. Subsequently, they fused a polydimethylsiloxane (PDMS) layer with the highly porous morphology of the nanofiber pads to form a charge trapping layer (CTL), further enhancing the power generation efficiency. Compared to simple planar surfaces, electrospun nanofiber friction surfaces offer the advantage of nanoscale roughness and inherent ferroelectricity. Moreover, by exploiting the charge trapping effect, they significantly improved the output performance and demonstrated its efficacy in inducing charge trapping effects in most nanofibers to the maximum extent.

Tayyab utilized an electrospinning method to fabricate PVDF nanofibers and incorporated commercially available printer ink (PI) nanofillers into the PVDF NFs to enhance their crystallinity further, thereby improving the overall performance of the PVDF-PI NF-based TENGs (Figure 11d) [111]. The study examined the performance of PVDF NFs and PVDF-PI NFs, which were developed for a period lasting five hours, by adjusting the tapping force on the TENG devices. Specifically, applying a force of 0.4 N to the PVDF NFs-based TENG yields a Voc of approximately 800 V and Isc of around 40 μA, respectively. By comparison, when subjected to an identical applied force of 0.4 N, the TENG based on PVDF-PI NFs, which were cultivated for a duration of 5 h, demonstrates Voc and Isc measurements of 1600 V and 130 μA, correspondingly (Figure 11e). Furthermore, the amplitude of β-phase enhancement in 5 h-grown PVDF-PI nanofibers reached its peak at 88%, leading to a maximum power output of 22 W/m^2^. This value exceeds that of PVDF nanofibers by more than two times, thereby establishing a new record for TENGs utilizing PVDF nanofibers. This suggests that using PI nanofillers can greatly improve the performance of PVDF-PI NFs, resulting in higher voltage and current output. This has an inspiring significance for the application of nanofillers in PVDF NFs.

Du and her team have proposed a new type of perovskite TENG containing a rhombic nanoarray with a CsPbIBr_2_/Pt/Co(OH)(CO_3_)_0.5_ friction surface (Figure 11f) [107]. Through meticulous manipulation of the structure of the friction surface and fine-tuning the dynamic properties of friction sets, a substantial augmentation in the density of friction charges on the surface of the friction layer has been successfully accomplished, resulting in an amplified electrical power density. The frictional density of charges has been raised from 5.34 nC/cm^2^ up to 9.51 nC/cm^2^. The combination of the bottom Co(OH)(CO_3_)_0.5_ layer as well as the added Pt layer has effectively minimized charge loss in the metallic electrode and expanded the inside charge area of friction charges within the friction layer. By implementing a three-layer structure consisting of Co(OH)(CO_3_)_0.5_/Pt/CsPbIBr_2_, the peak power density has been enhanced by a factor of 4.5, approaching 2.04 W/m^2^ when the contact point frequency is 0.5 Hz. The perovskite TENG has exhibited remarkable stability, enduring over 1350 cycles of ambient atmospheric circulation tests. The presented research offers novel insights into the advancement of TENGs utilizing perovskite or other semiconductor characteristics. These findings present promising opportunities for the use of TENGs in energy capturing and the creation of self-powered devices. 

He et al. accomplished the creation of a Schottky-junction-based TENG using a combination of conductive silver nanowires (AgNWs) and manganese-doped perovskite oxide (Bi_0.5_Na_0.5_)TiO_3_-BaTiO_3_(Mn-BNT-BT) nanostructures as a frictional negative layer for electrospun PVDF-HFP nanofibers [108]. The synergy between the perovskite oxide nanocrystals and the conductive metal nanowires improved the electrical performance of the electrospun fabric pad, increased the productive capacity of the PVDF-HFP fiber pad, and enhanced the electrical conductivity of the TENG. The optimized TENG achieved a remarkable 386% increase in output power compared to the original PVDF-HFP nanogenerator. For the mixed TENG containing 5% AgNWs and 5% Mn-BNT-BT nanocrystals, a peak Voc of 2170 V and a power of 18.91 mW were attained (Figure 11g,h), exceeding the operational efficiency of existing PVDF-based TENGs. Furthermore, the mixed fiber pad exhibited excellent durability as the friction negative layer in various environmental conditions. This Schottky-junction-based TENG has great potential in the fast-paced information age. It can be used as a recyclable, biodegradable, and wearable power supply for applications in physiologic surveillance, human–machine interface, and healthcare.

**Table 1:** Performance modulation results of TENG based on ferroelectric materials.

Regulatory Measures	Operating Mode	Device Dimension	Key Ferroelectric Materials	Voltage	Current/Current Density	Output Power/Power Density	Ref.
Surface modification and doping	CS	5 cm^2^	β-phase content (88%) PVDF	1350 V	0.5 mA	24 W/m^2^	[96]
	CS	50 × 50 mm^2^	PVDF/Ti_3_CNT_x_ films	152 V	20.1 mA/m^2^	2.5 W/ m^2^	[92]
	CS	4 inch^2^	NPZT nanoparticles	56 V	—	—	[94]
Surface coating	CS	40 × 40 mm^2^	GnPs@PVDF/PEEK	470 V	105 μA	11.8 mW	[93]
	CS	—	PVDF melt	16.1 V	53.1 nA	1.05 mW/cm^2^	[98]
Surface patterning	CS	20 × 20 mm^2^	pyramid-shaped PVDF	—	—	92.2 μW	[95]
	SE	44 × 25 mm^2^	micro-patterned BaTiO_3_@Ecoflex nanocomposites	319 V	9 mA/m^2^	2.9 W/m^2^	[100]
Porous structure	CS	35 × 35 mm^2^	porous aerogels of P(VDF-TrFE)	105.6 ± 10.8 V	—	62.8 mW/ m^2^	[101]
	CS	10 × 20 mm^2^	β-phase porous PVDF aerogel	90 V	15 mA	6.15 W/m^2^	[102]
	CS	23 × 23 mm^2^	porous electrospun PVDF mat	130 V	12 μA	3.5 W/m^2^	[103]
Micro- and nanoscale engineering	CS	40 × 40 mm^2^	ZnO@ZnSnO_3_/ZnSnO_3_/PDMS composite film (ferroelectric nanocubic ZnSnO_3_)	218 V	14.2 μA	24.625 μW/cm^2^	[27]
	CS	40 × 40 mm^2^	BaTiO_3_/PDMS composite (BaTiO_3_ ferroelectric nanocrystals)	375 V	6 μA	2.25 mW	[109]
	CS	25 × 25 mm^2^	PVDF-TrFE nanofiber mat	880 V	3.75 mA	—	[110]
	CS	30 × 30 mm^2^	PVDF-PI nanofibers	1600 V	130 μA	22 W/m^2^	[111]
	CS	—	Co(OH)(CO_3_)_0.5_/Pt/CsPbIBr_2_ multilayer nanoarray	243 V	3.1 μA/cm^2^	2.04 W/m^2^	[107]
	CS	20 × 20 mm^2^	PVDF-HFP + Mn-BNT-BT + AgNWs composite fiber mat	2170 V	—	47 W/m^2^	[99]

### 4.3. The Role of Environmental Factors

The distinctive characteristics of ferroelectric materials are widespread, and environmental variables like light [112,113] and temperature [114,115] have an obvious effect on structural and charge distribution changes in these materials. Ferroelectric materials’ characteristics can be directly impacted by changes in these variables. Consequently, we will go into great detail about how temperature and light affect the characteristics of TENGs made of ferroelectric materials in this section. This will provide deeper insights into the tuning and optimization performance of TENGs as well as a better understanding of how various environmental elements affect the behavior of ferroelectric materials.

#### 4.3.1. Temperature Change

Temperature is a key factor influencing the performance of ferroelectric materials and their performance in TENGs. This influence arises from the electrical properties of ferroelectric materials, particularly their polarization characteristics, which are highly dependent on ambient temperature. In extreme high- or low-temperature environments, the basic electrical properties of ferroelectric materials, such as dielectric constant and polarization strength, may undergo significant changes [114,115]. These changes directly impact the core function of TENGs—charge generation and accumulation capability. Therefore, researching the performance variations of TENGs based on ferroelectric materials under temperature changes is crucial. This innovation greatly improves the usability and effectiveness of TENGs that rely on ferroelectric materials for harvesting environmental energy and applications in sensing, ensuring high efficiency and stable performance across a wide temperature range, but also expands its prospects for applications in various temperature environments.

**Figure 12:**
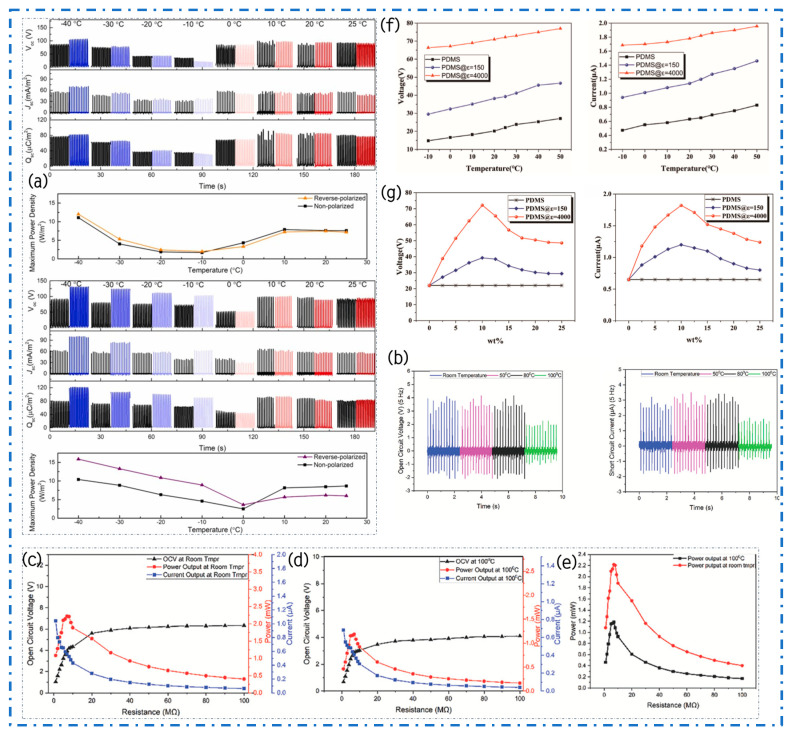
(**a**) Based on non-polarization (black) and counter-polarization (colored), the opening-circuit voltage, short-circuiting current density, short-circuit protection charge density, and max power generation of CFE and CTFE TENGs were measured over a temperature range of 25 °C to −40 °C. Reprinted with permission from Ref. [116] Copyright 2023 Elsevier Ltd. (**b**) Voc, (**c**) Isc, and (**d**) power output of the PGr-5 TENG at multiple degrees. (**e**) The maximum power output of PGr-5 was measured at both ambient temperature and 100 °C. Reprinted with permission from Ref. [117] Copyright 2024 Wiley-VCH GmbH. (**f**) Changes in VOC and ISC in PDMS@ε = 4000 at different temperatures. (**g**) Effects of different volume fractions of BaTiO_3_ (ε = 4000) on Voc and Isc. Reprinted with permission from Ref. [31] Copyright 2021 Elsevier Ltd.

In the study conducted by Ong et al., PVDF terpolymer blends of CFE and CTFE served as the frictional positive electrode layer, while SR acted as the frictional negative layer, aiming to investigate the performance of these materials under varying temperature conditions. The research spanned a temperature range from 25 °C to −40 °C, involving measurements of voltage and power output in both non-polarized (depicted by black curves) and polarized (depicted by colored curves) states of CFE and CTFE [116]. Analysis of the data plots reveals that (Figure 12a), when employing non-polarized CFE and CTFE, the TENG output initially decreases with decreasing temperature until reaching its nadir at approximately −10 °C and 0 °C, respectively. Subsequently, as the temperature descends further to −40 °C, there is a notable increase in both electrical power and output. Moreover, a comparison between outputs in non-polarized and polarized states indicates an escalation in the magnitude of piezoelectric enhancement with decreasing temperature. Initially, when the temperature decreases, the results of non-polarized and polarized materials show similar patterns, suggesting the absence of piezoelectric polarization. The discrepancy in output between non-polarized and polarized samples remains insignificant until the TENG output reaches its lowest point. However, subsequent to reaching this nadir, the difference in output between non-polarized and polarized samples gradually amplifies with further temperature decline. Particularly, at −40 °C, the output of polarized samples experiences a considerable enhancement, with the peak output of polarized CFE recorded at 105 V, 12 W/m^2^, 80 μW/m^2^, and 70 mA/m^2^, whereas that of polarized CTFE stands at 130 V, 16 W/m^2^, 120 μW/m^2^, and 115 mA/m^2^. Clearly, polarized CTFE offers a greater improvement in performance compared to CFE, especially with a temperature of −40 °C. At this temperature, the average enhancement rate of CTFE is 60%, which is much higher than CFE’s about 17%. This discovery implies that in cold situations, CTFE is more efficient than CFE in enhancing the output performance of TENGs. As a result, it becomes more suited as an alternate or extra electricity source for small electronic appliances in freezing temperatures. This study yields significant insights into the utilization of PVDF terpolymer blends under extreme temperature conditions and paves the way for their potential application in low-temperature energy harvesting and sensing technologies.

In a recent study, Sadeque and colleagues successfully fabricated graphene-integrated PVDF nanocomposite fibers using a hot stretching process and employed them in the production of TENGs [117]. The experimental findings illustrate that the addition of 5% graphene to PVDF fibers markedly improves their electrical performance, resulting in a respective increase of 1.41 times and 1.48 times in Voc and Isc. Considering that wearable devices are typically used outdoors and are exposed to varying environments ranging from extreme cold to extreme heat, the rapid temperature changes pose high demands on the performance stability of TENGs. Consequently, the research team conducted experiments on the PGr-5 fiber frictional electricity output utilizing a bespoke, motor-driven thread device within a specially designed enclosed heating chamber to assess its performance across a broad temperature spectrum. The experiment determined that the performance of PGr-5 fibers remained stable within the temperature range of 20 °C to 80 °C (Figure 12b–d). However, with a further elevation in temperature to 100 °C, there is a notable decline in output power. More precisely, when comparing the highest possible power draw of 2.2 mW at ambient temperature, the power output decreases approximately 46% when the temperature is raised to 100 °C (Figure 12e). This decline primarily stems from heightened thermal fluctuations at elevated temperatures, necessitating greater energy for electrons to surmount energy barriers. Conversely, under low-temperature conditions, insufficient electron energy prevents them from surpassing these barriers, resulting in a stable output of the PGr-5 TENG across a wide temperature range. These research findings underscore that graphene-integrated PVDF nanocomposite films not only amplify frictional electricity output but also exhibit long-term stability under extreme and fluctuating temperature conditions, rendering them an optimal choice for wearable devices in adverse weather conditions.

In recent research, Zhang and colleagues have devised an innovative method for fabricating high-dielectric materials that are resilient to fluctuations in environmental temperature, employing BaTiO_3_-based ceramic powders (ε = 4000). This pioneering material, when combined with PDMS, yields a high-performance composite film, serving as the frictional electric layer within TENGs [31]. Referred to as C-TENG, this composite film exhibits remarkable electrical stability across a broad temperature spectrum (−10 °C to +50 °C), with electrical stability confined to 14.6% and 14.8% (Figure 12f), a performance markedly superior to that of conventional PDMS film-based triboelectric nanogenerators (P-TENG). C-TENG displays Isc and Voc values of 1.82 μA and 72.2 V (Figure 12g), respectively. This superior electrical performance enables the C-TENG to illuminate 53 LED lights or power a calculator, thereby fully demonstrating its potential as a self-sustaining device. This investigation not only propels TENG technology forward from a materials science standpoint but also substantially diminishes device sensitivity to environmental temperature fluctuations, thereby broadening its potential applications across diverse environmental settings, including outdoor environments and variable climate conditions, as a viable power solution.

#### 4.3.2. Light Factor

Light exposure significantly influences the performance of ferroelectric materials in TENGs, primarily due to the triboelectric–photovoltaic coupling effect. This coupling integrates the triboelectric and photovoltaic effects; light exposure excites electrons and enhances the material’s electrical conductivity, thereby increasing TENGs’ power output capability [112,113]. This phenomenon not only provides new opportunities for designing and improving TENGs, but additionally encourages the use of photosensitive materials for electric power conversion and storage. This fosters the development of creative concepts and tactics for future energy technologies.

**Figure 13:**
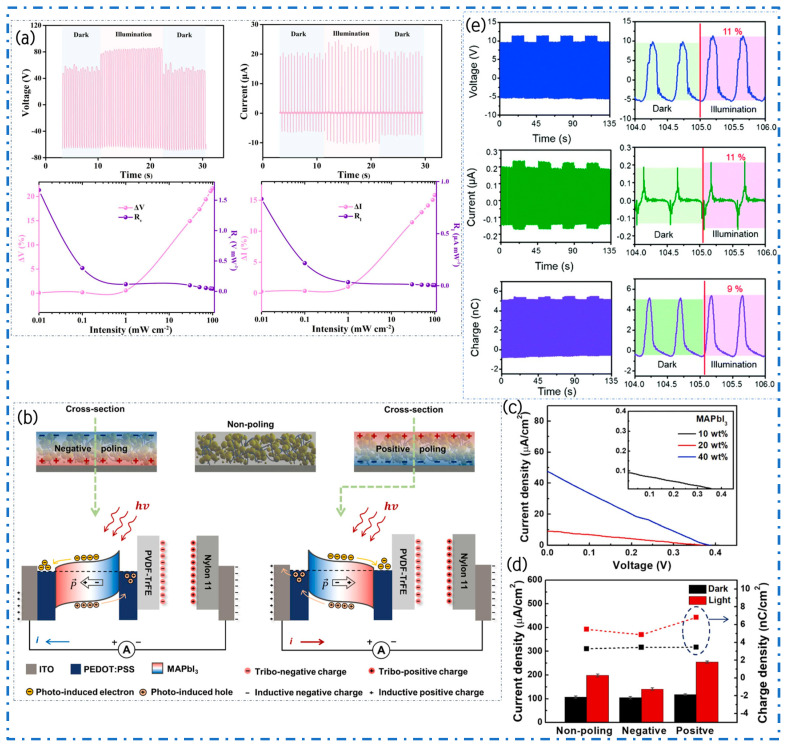
(**a**) Demonstrated the variation of TENG output performance using CBI/PDMS under light conditions. Reprinted with permission from Ref. [113] Copyright 2023 Elsevier Ltd. (**b**) The schematics as well as energy band diagrams of composite layers spanning different polarizations illustrate the separation of photogenerated carriers. (**c**) This article analyzes the current–voltage characteristics of the composite material that consists of different proportions of MAPbI_3_. (**d**) A comparison is made between the output current density and charge density of TENGs with non-poled, negatively poled, and positively poled composite layers, both in the presence and absence of light irradiation. Reprinted with permission from Ref. [118] Copyright 2023 Elsevier Ltd. (**e**) Under varying light conditions, the time-dependent values of the Voc, Isc, and charge amount (Q) of the photoenhanced TENG were measured, along with corresponding enlarged views at the moment of illumination. Reprinted with permission from Ref. [119] Copyright 2016 Royal Society of Chemistry.

The research team led by Wei and colleagues introduced the use of the low-toxicity, without-lead-halide perovskite CsBi_3_I_10_ (CBI) material in TENGs for the initial time. They designed a CBI-based light-enhanced TENG based on its broad light absorption characteristics [113]. In order to examine the precise impact of the friction–electro–optical coupling on the electrically generated efficiency of CBI/PDMS TENGs, the academic team carried out a set of comparison experiments in both low-light and well-lit conditions. The experimental results showed that illumination greatly improved the final result efficiency of the TENGs (Figure 13a). Upon activation of the light source, the positive output voltage immediately increased by approximately 21.7%. Additionally, when the CBI film was in the illuminated state, the output current exhibited an increase of about 15.8%. It is noteworthy that the enhancement in voltage and current quickly returned to their initial states after the end of illumination. This performance enhancement was observed only under illuminated conditions, while the output signal in the dark state was solely driven by the mechanical energy provided by the linear motor, further confirming that the performance enhancement mainly originates from the light-enhanced effect. This paper presents a novel approach for utilizing free-of-lead perovskite materials in TENGs. Additionally, it suggests a combination TENG design that takes advantage of the friction–electro–optical coupling effect to effectively harvest both mechanical and light energy.

Kim and colleagues have recently developed a composite material comprising PEDOT:PSS, PVDF-TrFE, and MAPbI_3_ particles for fabricating a novel TENG [118]. The primary advantage of this composite material lies in the inclusion of MAPbI_3_ particles, capable of generating photogenerated charge carriers when exposed to light. These charge carriers, facilitated by the effective transport pathways constructed by PEDOT:PSS, are directed to the electrodes, and the TENG’s output density of current is consequently greatly increased. The combination of materials is formed by encapsulating MAPbI_3_ components within PVDF-TrFE. This serves the purpose of not only preventing heat degradation but also improving the long-term sustainability of the material. Furthermore, through the polarization of MAPbI_3_ particles, the efficient separation of electron–hole pairs is facilitated, further amplifying the output density of current of TENGs. The experimental results indicate that an integrated film consisting of 40 weight percent MAPbI_3_ exhibited the maximum density of electricity output when exposed to light (Figure 13c). Under light irradiation, the ferroelectric properties of MAPbI_3_ (Figure 13b) result in an increasing output of TENGs as follows: negative polarization, non-polarization, and positive polarization. Notably, TENGs with positively polarized composite layers exhibit a 115% enhancement in current density and a 97% increase in charge density (Figure 13d). This remarkable performance enhancement is attributed not only to the material’s inherent photovoltaic properties but also to the optimization of charge carrier dynamics and stability in the design of composite material.

The recent publication by Su et al. introduces an innovative TENG based on lead MAPbI_3_ perovskite material, showcasing a distinctive light-enhanced operational mechanism [119]. This TENG leverages the inherent photoelectric properties of perovskite materials alongside their triboelectric effect, resulting in a synergistic enhancement of performance under sunlight exposure. When subjected to sunlight, the perovskite-based composite film demonstrates a significant increase in both photoconductivity and surface friction charge density, leading to a notable improvement in electrical output. Under full sunlight conditions, the TENG exhibits an 11% increase in open-circuit voltage (Voc), an 11% increase in short-circuit current (Isc), and a 9% increase in charge quantity (Q) (Figure 13e). These findings not only offer fresh insights into the application of perovskite materials within the realm of TENGs but also furnish valuable experimental support for amalgamating photoelectric and triboelectric effects to further augment the performance of TENGs.

## 5. Application Prospect of Ferroelectric Materials in Various Types of TENGs

By means of optimization of structural elements and other techniques, TENGs based on ferroelectric materials can have their properties fine-tuned, allowing them to perform more outstandingly in a variety of domains. The possible uses of ferroelectric material-based TENGs in multifunctional integration, self-powered electricity systems, and healthcare detection will be covered in the sections that follow.

### 5.1. Microenergy and Self-Powered Sensors

**Figure 14:**
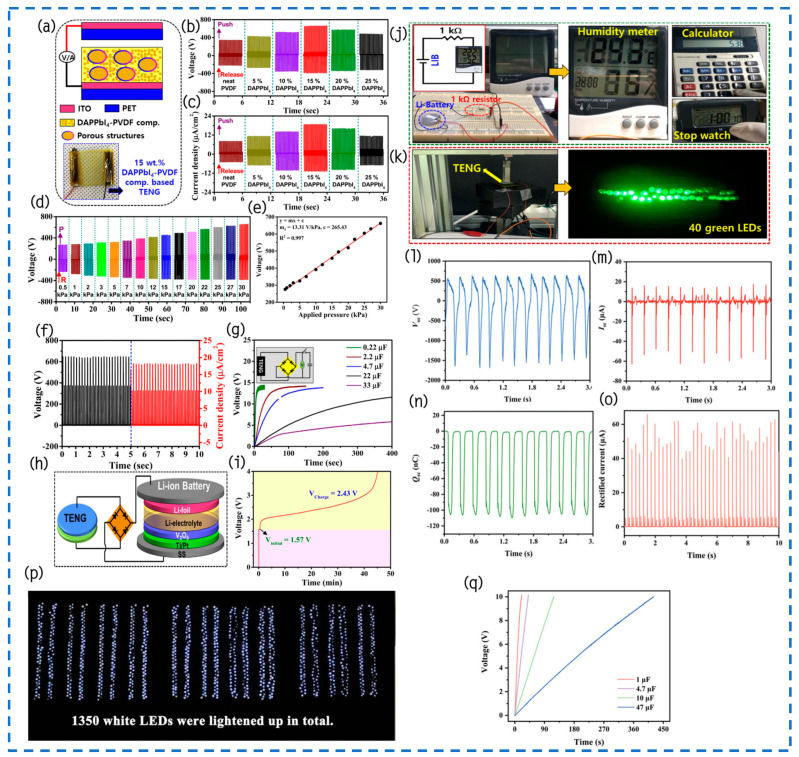
(**a**) An illustration of the 15 wt.% DAPPbI_4_-PVDF TENG. (**b**) and (**c**) the current density and output voltage of DAPPbI_4_-PVDF alloys prepared using TENGs with various proportions of DAPPbI_4_ perovskite are shown. (**d**) The output voltage of the 15 wt.% DAPPbI_4_-PVDF TENG is pressure-dependent. (**e**) The respective graph showing the sensitivity to pressure. (**f**) The current density and rectified output voltage of the 15 wt.% DAPPbI_4_-PVDF TENG were measured. (**g**) How different capacitors behave when charged using the 15 wt.% DAPPbI_4_-PVDF TENG’s output. (**h**) A model for the TENG charging circuit of a battery made of lithium ion. (**i**) Lithium-ion battery voltage dispersion when charged with 15 wt.% DAPPbI_4_-PVDF TENG. (**j**) The charged lithium-ion battery powers the temperature meter, timer, and calculator. (**k**) Using the mechanically stimulated power generation of the 15 wt.% DAPPbI_4_-PVDF TENG, illuminating forty commercial green LEDs. Reprinted with permission from Ref. [120] Copyright 2022 Elsevier Ltd. (**l**–**n**) Voc, Isc, and Qsc derived from SE-TENG based on PZT/GFF. (**o**) A photo demonstrating 1350 white LEDs being illuminated. (**p**) Current output by a bridge rectifier with a full waveform after correction. (**q**) A manual pressing of the SE-TENG produced a charges curve for commercial capacitors. Reprinted with permission from Ref. [121] Copyright 2021 Wiley-VCH GmbH.

The work conducted by Ippili et al. resulted in the successful development of a new, productive, and mechanically durable TENG based on DAPPbI_4_-PVDF [120]. This was achieved by incorporating DAPPbI_4_, a two-dimensional layered perovskite material with air stability, into PVDF (Figure 14a). Addition of 15 wt.% DAPPbI_4_ to the PVDF increased the proportion of the electrically active β-phase to approximately 90%, while significantly enhancing the dielectric constant and piezoelectric coefficient to approximately 47.4 and 26.6 pm/V, respectively. The composite material exhibited remarkable performance in the field of the TENG’s productivity (Figure 14b–e). It was able to produce voltages of up to approximately 662 V and current densities of around 18.7 µA/cm^2^. Additionally, it displayed a density of power roughly 4.28 mW/cm^2^ and a pressure sensitivity of about 13.31 V/kPa. Furthermore, the material demonstrated excellent mechanical toughness and reliability during operation. As a way to authorize the usefulness of this TENG as a direct current (DC) power source, the research team linked the identical 15 wt.% TENG to a full-amplitude bridge rectifier and subjected it to testing at 30 kPa/5 Hz. Test findings indicated that the rectified DC output efficiently stored energy in capacitors of varying capacities (Figure 14f,g). Additionally, the TENG demonstrated the ability to charge lithium-ion batteries (LIBs), reaching a charging rate significantly faster than other reported nanogenerators, charging LIBs to 4 V in just 45 min (Figure 14h,i). The trials additionally covered the connection of fully charged lithium-ion batteries (LIBs) to a 1 kΩ load resistor in order to supply power to tiny gadgets which included hygrometers, calculators, and stopwatches (Figure 14j). All these devices were successfully powered by the LIBs, confirming their reliability as energy sources. Furthermore, continuous mechanical driving of the 15 wt.% DAPPbI_4_-PVDF TENG, with snapshots and videos recorded during power supply, confirmed its immediate ability to drive commercial LEDs, further demonstrating the potential of the TENG as an energy harvester capable of self-charging power units, supporting the creation of wearable and easily transportable electronic goods with robust technical backing (Figure 14k). These research findings not only demonstrate the efficient energy conversion capability of TENGs based on DAPPbI_4_-PVDF composite material but also offer new possibilities for the future wider application of wearable electronic devices.

Recent research by Zheng et al. created an outstanding performance SE-TENG with a layer of dielectric made of an inorganic ferroelectric thin sheet with graphite fluoride (GFF) used as the friction material [121]. The design of the device optimizes the synergistic effect between frictional conductivity and dielectric properties, significantly enhancing its output performance. Specifically, the SE-TENG based on lead zirconate titanate (PZT)/GFF demonstrates outstanding electrical outputs, capable of generating up to 102.9 µC/m^2^, 1640 V, and 59.05 mA/m^2^, surpassing similar devices (Figure 14l–n). To demonstrate the practicality and potential applications of this novel TENG, the research team showcases its ability to power various electronic devices. The SE-TENG successfully illuminates 1350 white LEDs when connected in series, demonstrating its strong power generation capability (Figure 14o). Additionally, a full-wave bridge rectifying circuit efficiently converts the device’s alternating current (Figure 14p) into straight current. Under operating conditions of approximately 10 N and 4 Hz, simply tapping the device enables charging of capacitors, as demonstrated by the clear capacitor charging curve in Figure 14q. This device can charge capacitors with capacities of 1, 4.7, 10, and 47 µF to 10 V within 21, 41, 119, and 421 s, correspondingly, confirming that it can charge effectively. This SE-TENG based on PZT and GFF has a high current density of 59.05 mA/m^2^, which means it can power a variety of small electronics reliably and sustainably. It also shows promise as a convenient source of power for future microelectronic devices.

### 5.2. The Field of Biomedical Science

**Figure 15:**
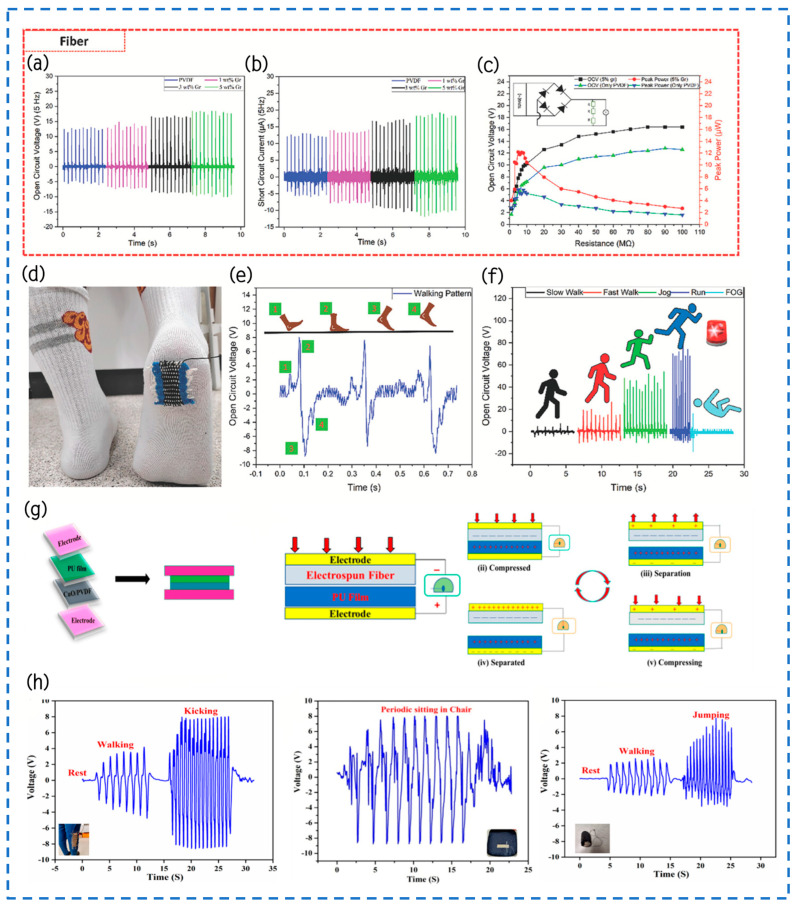
(**a**) Voc and (**b**) Isc of both the purity and PGr-(1–5%) TENG fibers were measured under specific conditions: a contact length of 3 cm and a frequency of 5 Hz. (**c**) The highest possible power generated by PGr-5 fibers can be measured by connecting solely PVDF fibers in an equivalent circuit. (**d**) The diagram displays the structure of the intelligent TENG socks, featuring TENG cloth affixed to the heel area. (**e**) The TENG has identified the walking mode. (**f**) The open-circuit voltage shows fluctuations across various velocities of human motion. Reprinted with permission from Ref. [117] Copyright 2024 Wiley-VCH GmbH. (**g**) Diagram illustrating the process of charge creation in the PVDF-CuO TENG. (**h**) The application of PC-8/PU TENGs in monitoring human movement and health status demonstrated their sensing performance. Reprinted with permission from Ref. [122] Copyright 2023 MDPI, Basel, Switzerland.

Sadeque et al. demonstrated the scalability in graphene-integrated PVDF nanocomposite fibers using a hot stretching technique, marking the first instance of such a demonstration [117]. The incorporation of 5% graphene into PVDF fibers resulted in a respective increase of 1.48 and 1.41 times in Isc and Voc (Figure 15a,b). As seen in Figure 15c, the PGr-5 fiber has an optimal power draw of 12 μW, which is 2.08 times more than that of the pure PVDF fiber. These fibers demonstrated exceptional stability under adverse environmental conditions, including alkaline mediums, varying temperatures, repeated washing, and extended use. To be able to put it into practice, the lower part of the sock’s heel area can be replaced with an extensible PGr-5 fabric gadget to confirm its incorporation with fibers. The sophisticated socks that were constructed were successfully utilized for analysis of gait during a whole stride cycle, which includes heel contact, toe connection, heel move, and toe lift (Figure 15d). As depicted in Figure 15e, the smart socks effectively identified all four motions in the walking period, showcasing remarkable sensitivity and selectivity. The intelligent sock has the ability to detect movements at different speeds, which may be detected through the alterations in the voltage that it outputs. While walking, the motion detector experiences a relatively low force, which leads to a decrease in the contact area and the output voltage. Nonetheless, when jogging or running, the force notably increases and thus provides a greater output voltage (Figure 15f). Thus, this sensor holds potential for investigating abnormal movement patterns associated with various neurological or orthopedic conditions, such as Parkinson’s disease, and for identifying muscle tension disorders and musculoskeletal irregularities in runners, thereby facilitating initial disease detection and subsequent rehabilitation processes.

Amrutha et al. have pioneered an innovative and adaptable TENG, using PVDF-CuO as the friction film with negative charge and polyurethane (PU) as the matching film with positive charge [122] (Figure 15g). Through a standardized load and frequency of 1.0 kgf and 1.0 Hz, an exhaustive examination of the TENG’s output voltage was executed employing tailored dynamic pressure settings. The empirical results demonstrate that the PVDF/PU arrangement alone produces a voltage output of just 1.7 V. However, a significant rise to 7.5 V is found when the CuO content increases, traveling from 2 wt.% towards 8 wt.%. Nevertheless, when the concentration of CuO is increased to 10 wt.%, there is a subsequent decrease in the generated voltage reaching 3.9 V. Drawing upon these experimental data, the research cohort showcased the utility of this optimized apparatus within the realm of real-time wearable sensors, proficient in monitoring human movement and health indicators, including respiration and heart rate. In particular, Figure 15h elucidates that, upon situating the friction sensor on denim, chairs, and footwear, respectively, it accurately detects and distinguishes numerous human movements, which could involve walking, leaping, sitting, and even kicking.

**Figure 16:**
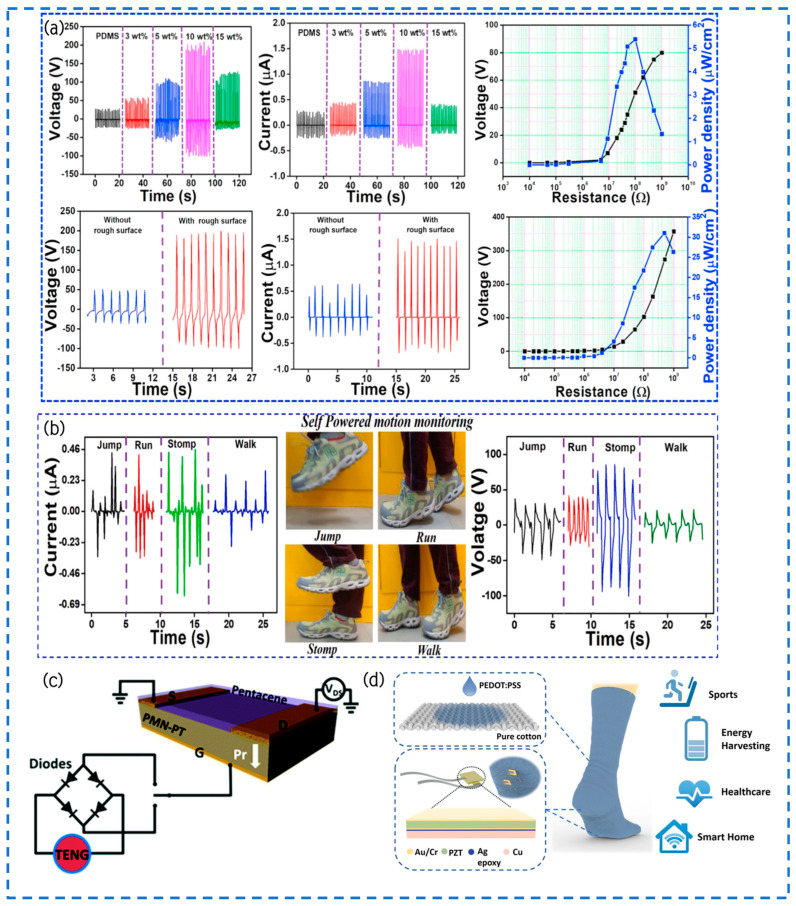
(**a**) The output performance of the TP-TENG. (**b**) A self-sustaining step tracking section utilizes a TP-TENG to measure and provide a signal for electricity following different physical actions. Reprinted with permission from Ref. [123] Copyright 2021 Elsevier Ltd. (**c**) The diagram illustrates the integration of a TENG with a ferroelectric gated field-effect transistor. Reprinted with permission from Ref. [124] Copyright 2015 Royal Society of Chemistry. (**d**) The self-sustaining sock is a wearable device that operates based on a schematic design and a certain functioning principle. Reprinted with permission from Ref. [125] Copyright 2019 American Chemical Society.

Sahu et al. successfully synthesized SCWO nanoparticles with a perovskite structure using the sol–gel method, leading to the invention of a revolutionary TENG (TP-TENG) [123]. The TENG comprises two friction layers: an aluminum layer and a polydimethylsiloxane composite film with a 10% perovskite structure (PDMS-SCWO) layer. The aluminum layer represents the positive friction layer, while the PDMS-SCWO layer represents the negative friction layer. When utilized with the vertical contact-separation method, the PDMS-SCWO composite film is capable of producing voltage responses upwards of 300 V and responses to current of 2.2 mA. The overall power density of the TP-TENG with a rough surface was measured to be 30.5 mW/cm^2^, significantly higher than that of the smooth-surfaced TP-TENG, which was only 5.5 mW/cm^2^ (Figure 16a). This notable improvement in performance is attributed to the enhancing effect of surface roughness on the frictional electric effect. Additionally, the TP-TENG exhibits exceptional flexibility and durability, rendering it highly suitable for real-time applications in sensing and analyzing various human gaits. To further investigate the energy conversion characteristics of the TP-TENG in the vertical contact-separation strategy, the scientific team attached the apparatus to the bottom of a shoe and performed a thorough analysis of the biomechanics of walking. The performance of the TP-TENG in daily activities was evaluated by imitating running, strolling, leaping, and trampling action (Figure 16b). The electrical signals generated by the device were monitored in real-time by connecting wires to an electrostatic meter, accurately recording voltage changes during the gait process. Specifically, when the heel lifts off the ground, the device registers a positive voltage peak; when the heel presses onto the device, a negative voltage peak is recorded. Analyzing the time difference between these positive and negative peaks enables a careful examination of the biomechanical characteristics of gait actions, offering crucial diagnostic insights for patients with gait disorders, which holds significant clinical and rehabilitative value.

### 5.3. The Multifunctional Integration of Ferroelectric Materials and TENGs

Recent research led by Fang and his colleagues introduced an innovative integrated module for self-powered ferroelectric transistor memory, seamlessly merging ferroelectric field effect transistor (FET) technology with TENGs (Figure 16c) [124]. The cornerstone of this advancement lies in a groundbreaking TENG design, comprising a self-assembled polystyrene nanosphere array and a porous polyvinylidene fluoride (PVDF) film. Such a remarkable performance leap establishes a robust groundwork for TENG’s application within the realm of self-powered memory. In addition, by utilizing this efficient TENG, the research team was able to effectively carry out direct polarization treatment upon 0.65Pb (Mg_1/3_-Nb_2/3_) O_3_-0.35PbTiO_3_ (PMN-PT) single crystals. This step is crucial in achieving the fundamental functioning of ferroelectric field-effect transistor (FET) memory components. They then used pentacene as the material for the channel, employed PMN-PT isolated crystals as an insulator for the gate, and carefully designed a ferroelectric field-effect transistor with a bottom-gate configuration. Rigorous systematic testing and evaluation validated the transistor memory component’s impressive on/off current ratio of approximately 10^3^, showcasing commendable electrical characteristics and storage performance. Moreover, the study successfully showcased the feasibility of altering the polarization state of the FET gate insulator through an ingeniously designed circuit, tapping into the TENG with a finger to encode information. This breakthrough not only signifies a technological milestone but also offers a fresh outlook and solution for the advancement of self-powered storage systems.

Zhu and colleagues conducted a study where they developed an innovative and revolutionary sock called the S^2^-sock (Figure 16d). This sock is capable of generating its own electricity and has several functions. The researchers achieved this by combining a fabric coated with PEDOT:PSS with a piezoelectric chip made of lead zirconate titanate (PZT) [125]. This innovation not only endowed the sock with fundamental energy harvesting capabilities but also facilitated the generation of characteristic waveforms. Consequently, it provides a straightforward and efficient means of detecting human walking patterns and tracking movements. Moreover, as a practical demonstration of sensor fusion, the combination of TENG and PZT sensors enabled the rapid assessment of sweat levels in the body through signal outputs resulting from mechanical contact. The enhanced multilayer PEDOT:PSS patterns and integrated thin PZT chips collaborated to achieve precise gait sensing and detailed analysis of contact forces. In essence, this technology effectively amalgamates the functionalities of two sensors, allowing them to synergistically mitigate potential drawbacks associated with their individual sensing mechanisms.

## 6. Summary and Future Outlook

This paper offers an overview of the basic principles underlying ferroelectric materials and the operational methods of TENGs. It then delves into the optimization of the performance of TENGs through the manipulation of material composition and structure. Furthermore, this article thoroughly analyzes the potential applications of ferroelectric materials in diverse fields such as energy, biology, medicine, and flexible electronics.

Ferroelectric materials possess a unique advantage over other materials in their concurrent ferroelectric and piezoelectric properties, allowing for charge separation and polarization under external electric fields or mechanical stress. These distinctive physical properties render ferroelectric materials highly versatile in applications such as sensors, energy storage devices, and capacitors. Going forward, research can concentrate on the following key areas:

(1) Material development and optimization: Advancing performance of TENG necessitates the development of novel ferroelectric materials, which can be combined with other materials to achieve enhanced functionality;
(2) Energy conversion efficiency: Although materials with high dielectric constants and piezoelectric coefficients have been identified, the effective conversion of mechanical energy into electrical energy still presents a considerable obstacle. Improving efficiency mandates the consideration of material properties, device structures, and external environmental factors like temperature and humidity;
(3) Mechanical and electrical coupling: In TENG devices, optimizing the coupling between mechanical and electrical elements is paramount to enhancing output performance. This necessitates meticulous design and a profound understanding of material properties to ensure the efficient transfer and accumulation of charges during stress and friction processes;
(4) Miniaturization and integration: While the potential for microdevice applications of TENGs based on ferroelectric materials is promising, challenges persist regarding miniaturization and integration. Addressing these issues requires tackling technological hurdles like material processing, device packaging, and interface compatibility with other electronic components.

To tackle these challenges, future research efforts should focus on exploring novel ferroelectric materials, refining device designs, developing more efficient energy conversion technologies, and enhancing the adaptability of devices to environmental conditions. By utilizing this approach, it is expected that TENGs utilizing ferroelectric materials will become increasingly important in various areas of energy collecting and sensing. The overall goal of this review is to offer readers a thorough comprehension and stimulate new research avenues.

## Data Availability

Data available on request from the authors.
